# Structure–Activity Study of the Antimicrobial Lipopeptide Humimycin A and Screening Against Multidrug-Resistant *Staphylococcus aureus*

**DOI:** 10.3390/antibiotics14040385

**Published:** 2025-04-05

**Authors:** Md Ramim Tanver Rahman, Louis-David Guay, Ismail Fliss, Eric Biron

**Affiliations:** 1 Faculty of Pharmacy, Université Laval, Québec, QC G1V 0A6, Canada; ramimbau@gmail.com (M.R.T.R.); louis-david.guay.2@ulaval.ca (L.-D.G.); 2 Laboratory of Medicinal Chemistry, CHU de Québec-Université Laval Research Center, Québec, QC G1V 4G2, Canada; 3 Institute of Nutrition and Functional Foods, Université Laval, Québec, QC G1V 0A6, Canada; ismail.fliss@fsaa.ulaval.ca; 4 Research Center in Infectious Diseases, Université Laval, Québec, QC G1V 0A6, Canada; 5 Department of Food Science, Faculty of Agriculture and Food Sciences, Université Laval, Québec, QC G1V, Canada

**Keywords:** antimicrobials, lipopeptides, chemical synthesis, antibiotic resistance, multidrug-resistant *Staphylococcus aureus*, bovine mastitis, alternative to antibiotics, humimycin

## Abstract

Background: The emergence of multidrug-resistant (MDR) *Staphylococcus aureus* presents a critical global health challenge due to treatment failures and high mortality rates. Faced with this growing threat, new antimicrobials with original modes of action are urgently needed, and antimicrobial peptides proved to be promising alternatives. Objectives: The aim of this study is to explore the structure–function relationship of the lipopeptide humimycin A, compare the spectrum of activity of the synthetic analogs against a panel of *S. aureus* isolates, and investigate their binding to the humimycin target, the lipid II flippase MurJ. Methods: Humimycin A and 15 analogs were produced by solid-phase peptide synthesis, and their antimicrobial activity was evaluated by agar diffusion and microtitration assays against 19 *S. aureus* isolates from bovine mastitis and other pathogens. Results: Among the synthesized peptides, four humimycin analogs exhibited activity against methicillin-sensitive and methicillin-resistant *S. aureus*, as well as several isolates in the panel, including MDR *S. aureus*, with minimal inhibitory concentration values ranging from 0.5 to 256 µg/mL. Results from the structure–activity relationship study showed that the β-hydroxymyristoyl lipid chain and C-terminal carboxylic acid are essential for antimicrobial efficacy. In presence of human erythrocytes, the active humimycin analogs exhibited moderate hemolytic activity, suggesting selectivity indexes ranging from 3 to 27 against the more sensitive *S. aureus* strains. Critical micelle concentration measurements elucidated micelle formation and proved to not be essential for the antibacterial activity. Molecular docking and 100 ns simulations with the lipid II flippase MurJ (PDB: 5T77) provided favorable binding energy. Conclusions: The findings underscore the potential of humimycin analogs as antimicrobials for preventing and treating MDR *S. aureus* infections in veterinary, animal husbandry, and human medicine.

## 1. Introduction

*Staphylococcus aureus* is a versatile commensal organism and a significant opportunistic pathogen, which is frequently isolated from superficial skin to life-threatening infections in both humans and animals [1,2]. Classified as an ESKAPE pathogen, the spread of multidrug-resistant *S. aureus* (MDRSA) such as methicillin-resistant *S. aureus* (MRSA) has propelled it as a high priority pathogen by the World Health Organization (WHO) for the development of new antimicrobials [3]. Severe MDRSA infections can lead to systemic inflammatory responses [4] and complications such as bacteremia-sepsis, endocarditis, pneumonia, osteomyelitis, septic arthritis, urinary tract infections, surgical site infections, and skin diseases, which are particularly challenging to treat and often require prolonged antibiotic therapy and surgical intervention [5,6]. In the case of MRSA bacteremia, a life-threatening bloodstream infection, the mortality rate exceeds 25% within 3 months of diagnosis [7], and survivors may experience significant morbidity and long-term health consequences.

Likewise, in livestock and food production, *S. aureus* poses a major challenge to the global dairy industry, particularly through bovine mastitis infections [8]. These infections reduce milk production and revenue, cause reproductive difficulties, and lead to increased expenses due to the culling of infected animals. Veterinary treatment costs, estimated at USD 147 per cow annually, combined with the elimination of tainted milk and the growing concern over antibiotic residues in milk, further intensify the economic and public health burdens associated with *S. aureus* in dairy farming [9]. Additionally, *S. aureus* in milk produces toxins that threaten food safety. Its virulence factors have been highlighted by the WHO as its capacity of persistence causes recurring infections, hindering dairy herd control and management [2]. Repeated exposure to antibiotics, often necessitated by these persistent infections, not only complicates treatment strategies, but also contributes significantly to the development of drug-resistant bacteria. This highlights the interconnectedness of *S. aureus* infections across human and animal health, underscoring a “One Health” approach in addressing its impact.

Presently, the treatment of MDRSA infections relies on a limited selection of antibiotics, with vancomycin (first FDA approval in 1958) and daptomycin (FDA approval in 2003) as the last-resort drug. Unfortunately, resistance to these drugs is emerging due to alterations in peptidoglycan synthesis in *S. aureus*, further narrowing the therapeutic choice [10]. The detection of vancomycin-intermediate and vancomycin-resistant *S. aureus* strains exemplifies the ongoing arms race between antimicrobial development and bacterial adaptation. Consequently, the search for new antimicrobial compounds that can skirt resistance mechanisms and effectively treat MDRSA infections is of paramount importance. The mechanisms by which *S. aureus* bacteria develop antibiotic resistance have been extensively studied and continue to evolve, complicating the implementation of new treatment strategies. The bacterium’s ability to acquire resistance genes and mutations, coupled with its versatile virulence factors, enables it to thrive in the face of antimicrobial pressures [11]. These highlights underscore the limitations of the current therapeutic options and emphasize the need for stringent infection control measures to combat these multidrug-resistant pathogens.

Antimicrobial lipopeptides (AMLPs) have emerged as a promising class of antimicrobial agents due to their unique structures and original mechanisms of action which differ from most currently used antibiotics. Most AMLPs primarily interact with negatively charged phospholipid bilayers of bacterial cell membranes. This interaction is facilitated by the amphiphilic nature of lipopeptides, which allows them to integrate into the lipid membrane and disrupt membrane integrity (through pore formation or carpet/detergent-like mechanism) [12] and interact with membrane proteins or intracellular components to disturb the essential cellular processes and lead to bacterial cell death [13,14]. The process of membrane lysis induced by AMLPs is typically physical, rapid, and irreversible. Moreover, AMLPs are less likely to promote resistance or cross-resistance and if resistance does occur, it develops more slowly compared to traditional antibiotics [15]. Taken together, these characteristics position AMLPs as promising scaffolds and leading tools in the fight against microbial resistance, with potential application in antibacterial [16], antifungal [17], and antiviral [18] therapies.

Among promising AMLPs of bacterial origin, humimycins show very attractive activities against *S*. *aureus*. Humimycins A **1** and B **2** are composed of a heptapeptide bearing an N-terminal *β*-hydroxy myristoyl lipid chain (Table 1). They show strong inhibitory activities against firmicutes, particularly the *Staphylococcus* and *Streptococcus* species with minimum inhibitory concentration (MIC) values ranging from 4 to 32 μg/mL [19]. A novel approach was employed to discover humimycins by combining bioinformatics and chemical synthesis. Rather than depending on bacterial cultures to discover and produce non-ribosomal peptide antibiotics like colistin [20] and vancomycin [21], this method used primary sequencing data from the human microbiome to predict natural antibacterial product structures and pinpoint humimycins, with a focus on *Rhodococcus equi* and *Rhodococcus erythropolis* [19].

The proposed mechanism of action of humimycins involves the inhibition of the lipid II flippase enzyme, MurJ, which is essential for translocating lipid II across the cytoplasmic membrane into the periplasm. This process is crucial for maintaining cellular integrity and morphology, rendering MurJ an attractive target for novel antibacterial agents. By inhibiting MurJ, humimycins disrupt the peptidoglycan biosynthesis pathway, leading to the cytoplasmic accumulation of lipid II substrates. This accumulation results in the inability to synthesize and repair the bacterial cell wall, ultimately triggering cell lysis. The disruption of this enzymatic pathway highlights the potential of targeting such mechanisms as a highly effective strategy in the development of new antimicrobial agents [22,23]. Several synthetic analogs such as humimycin W **3** (Y1W) and humimycin 17S **4** (Y1W, y3w, F4W) have been reported (Table 1) and have demonstrated activities against several MDRSA strains including MRSA USA300 [22,24]. The results showed that the substitution of Tyr and Phe residues by Trp is not detrimental and can improve the activity in humimycin analogs. Additionally, humimycin 17S **4** has been shown to potentiate the activity of *β*-lactam antibiotics (e.g., dicloxacillin and carbenicillin) without leading to notable resistance in *S. aureus* [22]. Given the promising mechanism of action and the potential of humimycins, the opportunity to design novel antimicrobial lipopeptides inspired us to explore the humimycin scaffold and develop new analogs with enhanced pharmacological properties.

In humimycin, the fatty acid moiety is a linear and saturated chain, which is a common structural feature among non-ribosomal lipopeptides [19]. However, the incorporation of mono- or polyunsaturated fatty acids, either cis or trans isomers, could yield analogs with potent antibacterial properties against various pathogens [25]. This approach is promising, as fatty acids themselves sometimes exhibit higher MIC values, and polyunsaturated fatty acids have demonstrated greater antimicrobial activity compared to their saturated counterparts [26]. Moreover, liposomal formulations of unsaturated fatty acids such as oleic acid have been shown to enhance antimicrobial activity while reducing the toxic side effects [27]. By appending unsaturated fatty acids to the heptapeptide core of humimycin, we hypothesized that the antimicrobial activity of the resulting analogs could be modulated. Transcriptional studies have revealed that unsaturated fatty acids like myristoleic acid can influence the expression of various biofilm virulence-related genes, including those encoding lipase and hyaluronate lyase [28]. Despite the potential benefits of incorporating unsaturated fatty acids in the development of analogs with improved activity, there is currently no clear structure–function relationship guidelines or consensus on the ideal fatty acid chain length for optimal antimicrobial activity. This research aims to address this gap by systematically evaluating a series of unsaturated fatty acids with varying chain lengths on the humimycin peptide scaffold to identify analogs with enhanced antibacterial properties. The stereochemistry of the β-hydroxyl group has also been shown to be important for humimycin A **1** as the *S* enantiomer is fourfold more active than its *R* counterpart, but it did not impact the activity of the humimycin W **3** [24] and 17S **4** [22] analogs. Therefore, we also aimed to investigate the importance of β-hydroxyl group’s stereochemistry for the activity of humimycin analogs. Additionally, it was also relevant to investigate C-terminal modification, as C-amidation has been shown to potentiate antibacterial activity in some cases, while minimizing hemolytic effects [29].

Here, we report on the chemical synthesis of humimycin A and a series of analogs, as well as the assessment of their antimicrobial activity against various bacterial pathogens, including a panel of *S. aureus* isolates from bovine mastitis. The structure-activity relationship study allowed us to identify essential residues in the humimycin scaffold for antimicrobial efficacy and identify synthetic analogs with activity against MDRSA strains. By examining their effects on growth curves and critical micelle concentration, we found that these humimycin analogs have promising properties and modes of action. Additionally, docking and molecular dynamics simulations were performed to evaluate the interactions between the most promising analog and the proposed humimycins target, the lipid II flippase MurJ.

**Table 1:** Humimycin A and synthetized analogs.

#	Peptide	Lipid Chain	Peptide Sequence							C-Term
			1	2	3	4	5	6	7	
**1**	Hum A [19]	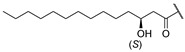	Tyr	Ser	d-Tyr	Phe	d-Thr	Val	Val	COOH
**2**	Hum B [19]					Tyr		Ile		
**3**	Hum W [24]		Trp							
**4**	Hum 17S [22]		Trp		d-Trp	Trp				
**5**	Hum A_isoC15	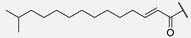								
**6**	Hum A_isoC13	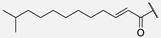								
**7**	Hum W_geranoyl	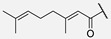	Trp							
**8**	Hum W_octanoyl	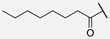	Trp							
**9**	Hum W_mandelic	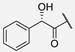	Trp							
**10**	Hum W_maleic	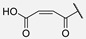	Trp							
**11**	Hum W_fumaric	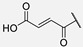	Trp							
**12**	Hum W1,3	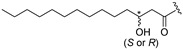	Trp		d-Trp					
**13**	Hum W1,3-A6		Trp		d-Trp			Ala		
**14**	Hum W1,3,5		Trp		d-Trp		d-Trp			
**15**	Hum W amide		Trp							CONH_2_
**16**	Hum W1,3 amide		Trp		d-Trp					CONH_2_
**17**	Hum W1,3-A6 amide		Trp		d-Trp			Ala		CONH_2_

## 2. Results

### 2.1. Synthesis of Humimycin Analogs

Humimycin A **1** and synthetic analogs **5**–**17** (Table 1) were prepared by solid-phase peptide synthesis (SPPS) using the Fmoc/tBu strategy. After cleavage from the solid support and side chains deprotection, each analog was purified by preparative HPLC and characterized by mass spectrometry (MS) (Table S1). In the case of analogs **12**–**17**, the (*R*) and (*S*)-enantiomers were isolated during the purification to elucidate the influence of the β-hydroxymyristoyl stereochemistry on the antimicrobial activity. The synthesized analogs were obtained with purities ranging from 85 to 95% and overall yields between 3% and 5% (Table S1, Figure S1).

### 2.2. Antimicrobial Activity of the Humimycin Analogs Against Gram-Positive and Gram-Negative Bacteria

**Figure 1:**
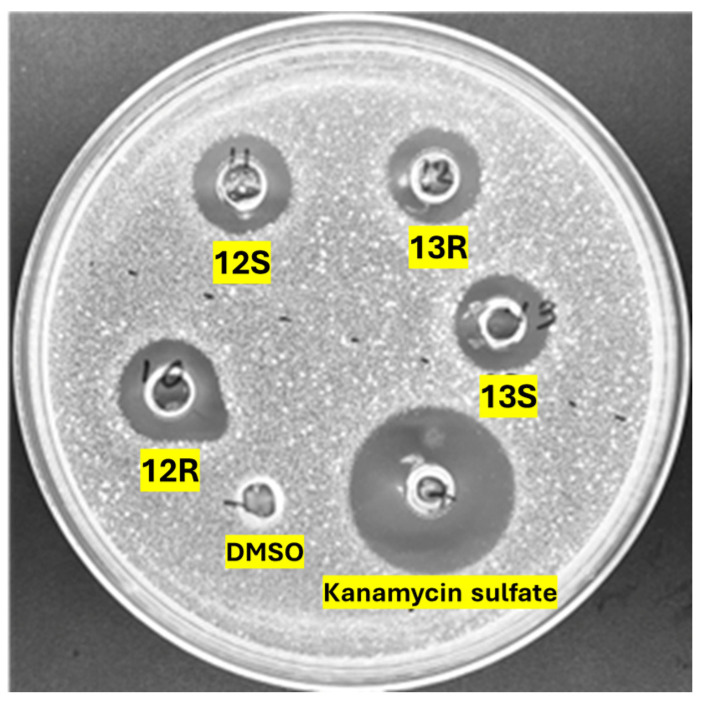
Antimicrobial activity of synthetic humimycin analogs **12*R***, **12*S***, **13*R*** and **13*S*** against *S. aureus* ATCC 29213 using an agar well diffusion assay. Inhibition zone diameters (mm) were, respectively, 17, 15, 15, and 15. Each agar well was loaded with 70 µL of a peptide solution (256 µg/mL). Kanamycin sulfate (concentration: 256 µg/mL, 24 mm) and 5% DMSO in water (0 mm) were used as the positive and negative controls, respectively.

The antimicrobial activity of the synthetic humimycin analogs **5**–**17** was first evaluated against *S. aureus* ATCC 29213 using the agar diffusion assay. Among the synthesized lipopeptides, only analogs **12*S***, **12*R***, **13*S***, and **13*R*** exhibited inhibitory activity against *S. aureus*, as evidenced by the clear zones of inhibition ranging from 15 to 17 mm (Figure 1) In contrast, the remaining analogs failed to exhibit any zones of inhibition (Figure S2). For comparison, the positive control kanamycin showed the largest zone (24 mm) of inhibition. In contrast, no zone of clearance was observed for the negative control (5% DMSO), confirming the absence of antibacterial activity. The agar diffusion assay results highlight the potential of the synthetic humimycin analogs **12** and **13** as antibacterial agents against the clinically relevant *S. aureus* strains.

Next, the antimicrobial activity of the humimycin analogs was evaluated against a panel of Gram-positive and Gram-negative bacterial strains, using microtitration assays to determine the minimum inhibitory concentration (MIC). As observed in the agar diffusion assay, only peptides **12*S***, **12*R***, **13*S***, and **13*R*** exhibited potent antibacterial activity against the methicillin-sensitive (MSSA) and methicillin-resistant *S. aureus* (MRSA) strains with MIC values ranging from 2 to 128 μg/mL (Table 2). No inhibition of Gram-positive *Bacillus subtilis* and Gram-negative *Escherichia coli*, *Pseudomonas aeruginosa*, *Salmonella enterica* was observed. Unfortunately, N-terminally modified analogs **5**–**11** bearing the different fatty acyl chains or dicarboxylic acids failed to exhibit antimicrobial activity against the tested bacteria at the highest concentration measured. The same results were obtained with the C-terminal amide analogs **15**–**17**. The structure-activity study showed that for analogs **12** and **13**, the stereochemistry of the β-hydroxyl group does not play a critical role for inhibition, as only a twofold difference in activity was observed between the (***S***)-isomers **12*S*** and **13*S*** and their (*R*)-isomer counterpart **12*R*** and **13*R***. Surprisingly, an important difference in the inhibitory activity of analogs **12** and **13** on the tested *S. aureus* strains was observed. While peptides **12*R*** and **12S** displayed similar MIC values against the MSSA and MRSA, a 32-fold decrease in activity was observed for peptides **13*R*** and **13*S*** against the methicillin-resistant strain. These findings highlight the importance of the lipid chain, peptide sequence, and β-hydroxyl group and its stereochemistry in modulating the antimicrobial activity of humimycin analogs and provide valuable insights for the development of potent antibacterial agents targeting both methicillin-sensitive and MDR *S. aureus* strains.

### 2.3. Antimicrobial Activity of the Humimycin Analogs Against S. aureus Isolates

With the promising results obtained on methicillin–oxacillin-resistant *S. aureus* ATCC 43300, the inhibitory activity of humimycin analogs **12*S***, **12*R***, **13*S***, and **13*R*** was next evaluated against a panel of 19 *S. aureus* strains isolated from bovine mastitis [30]. Among these strains, eleven strains were sensitive to antibiotics, one was resistant to only tetracycline (TET) and seven were multidrug resistant, necessitating the exploration of alternative antimicrobial options. The results showed great variability in the effectiveness of the analogs against both antibiotics sensitive and resistant strains with MIC values varying from 0.5 to 256 µg/mL (Table 3).

Five strains (4-131, 4-133, 4-134, 4-162, and 4-165) were resistant to the humimycin analogs at the tested concentration of 256 µg/mL. While *S. aureus* strains 4-131 and 4-162 were sensitive to antibiotics, strains 4-133, 4-134, and 4-165 exhibited multidrug resistance. The effectiveness of the humimycin analogs against fourteen *S. aureus* strains, including five antibiotic-resistant strains, is particularly notable. The highest activity was observed against MDR *S. aureus* strain 4-171 for analogs **12*R***, **12*S***, and **13*R***, with MIC values of 2.0, 1.0, and 0.5 µg/mL, respectively. Overall, except for isolates 4-121, 4-138, and 4-171, the synthetic analogs and their stereoisomers demonstrated similar activities across the tested strains, with no or two-fold change in the MIC values. *S. aureus* strain 4-134, with resistance to 11 antibiotics, remains the most broadly resistant isolate in our panel.

The varying efficacy of different humimycin analogs against specific resistant strains emphasizes the need for targeted therapeutic approaches. Personalizing treatment based on the resistance profile of the infecting strain could potentially improve clinical outcomes by selecting the most effective humimycin analog or combination therapy. Further investigations are required to elucidate the underlying mechanisms of cross-resistance and to develop strategies to overcome these challenges. Understanding the molecular basis of resistance could facilitate the rational design of next generation humimycin analogs with an improved activity against resistant strains, thereby expanding the therapeutic potential of this promising class of antimicrobial lipopeptides.

### 2.4. Impact of Humimycin Analogs on Bacterial Growth

**Figure 2:**
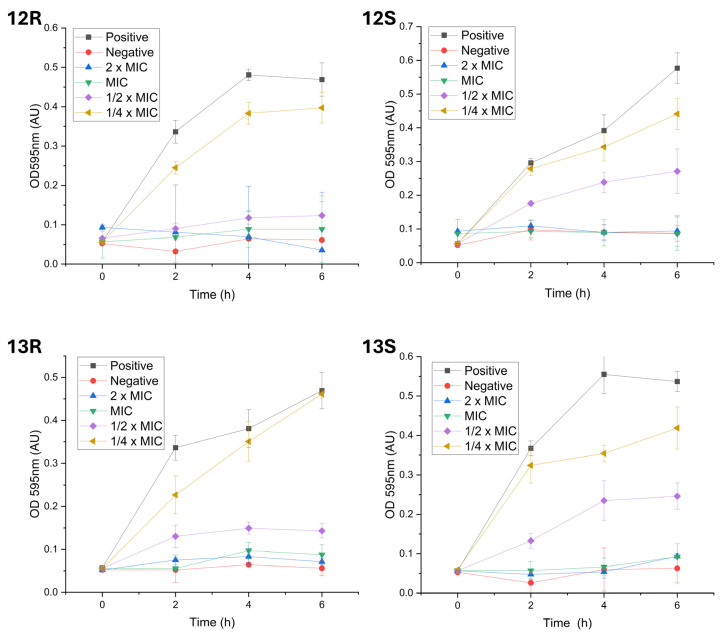
Growth kinetics of *S. aureus* ATCC 29213 in the presence of humimycin analogs **12*R***, **12*S***, **13*R***, and **13*S***. Cultures of *S. aureus* were treated with various concentrations (2 × MIC, MIC, ½ × MIC, and ¼ × MIC) of the peptides and their growth was monitored by measuring the optical density at 595 nm. The positive control consisted of an untreated *S. aureus* culture, while the negative control contained only the growth medium without bacterial inoculation. The data represents the mean ± standard deviation of three independent experiments.

Understanding how bacteria adapt to different environmental conditions and exposure to antimicrobials has become an essential step in the development of effective therapeutics, particularly in the face of the growing challenge of antibiotic resistance. The impact of humimycin analogs on bacterial growth was evaluated by analyzing growth kinetics of *S. aureus* ATCC 29213 exposed to the compounds. Following incubation, *S. aureus* entered the exponential phase at 2 h and reached the stationary phase between 4 and 6 h (Figure 2). Humimycin analogs **12*R***, **12*S***, **13*R***, **13*S*** at their MIC effectively inhibited the growth of *S. aureus*. This inhibition was observed as the cultures entered the exponential growth phase, approximately 2 h post-inoculation. The optical density (OD) measurements of the cultures treated with these analogs were significantly lower than those of the positive control and closely approximate to the values of the negative control. Furthermore, when *S. aureus* was exposed to half the MIC of the analogs, the growth patterns were similar to those of the positive control. The rapid growth inhibition observed suggests that all analogs tested have inhibitory effects on *S. aureus* at MIC or higher concentrations, indicating that these compounds may disrupt crucial cellular processes necessary for bacterial survival and replication.

### 2.5. Hemolytic Activity

**Figure 3:**
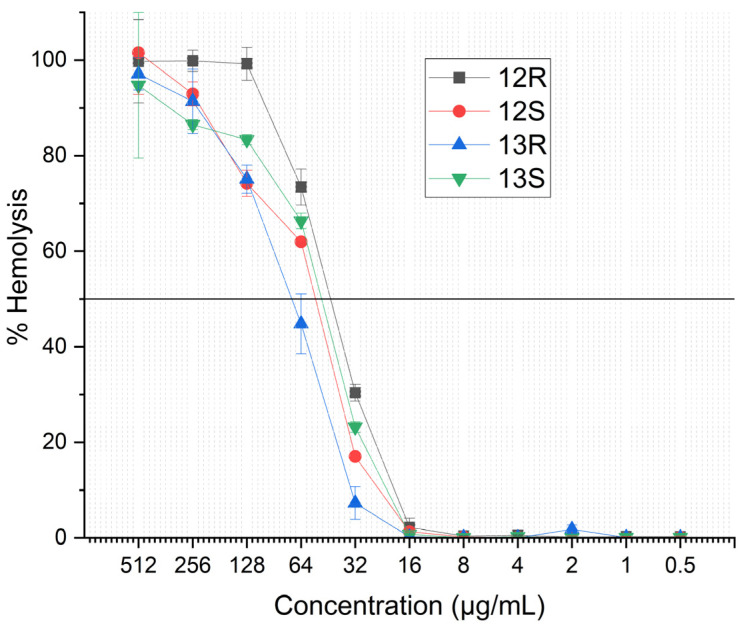
The hemolytic activity of synthetic humimycin analogs **12*R***, **12*S***, **13*R***, and **13*S*** on human erythrocytes. Positive control: 0.2% Triton X-100 in PBS, negative control: PBS. The data represent the mean ± standard deviation of three independent experiments.

Several lipopeptides have been shown to exert considerable cytotoxicity. To evaluate the potential undesired effects of humimycin analogs on eucaryotic cells, the hemolytic activity of the synthetic lipopeptides was assessed on human red blood cells (RBC). The results showed that humimycin analogs **12*R***, **12*S***, **13*R***, and **13*S*** cause considerable hemolysis with HC_50_ values of 44.8, 60.8, 76.8, and 48 µg/mL, respectively (Figure 3). This suggests a selectivity index ranging from 3 to 24 against *S. aureus* ATCC 29213. However, for resistant strains, the selectivity index is reduced to 2 or less when compared to the MIC values from the previous section. Among the tested peptides, analog **13*R*** showed the highest selectivity index for most tested strains sensitive to humimycin analogs. We hypothesize that the important hemolytic activity of humimycin analogs could be due to high hydrophobicity of the peptides, as it has been shown that hydrophobicity in peptides can correlate with hemolytic activity [31].

### 2.6. Determination of the Critical Micelle Concentration

**Figure 4:**
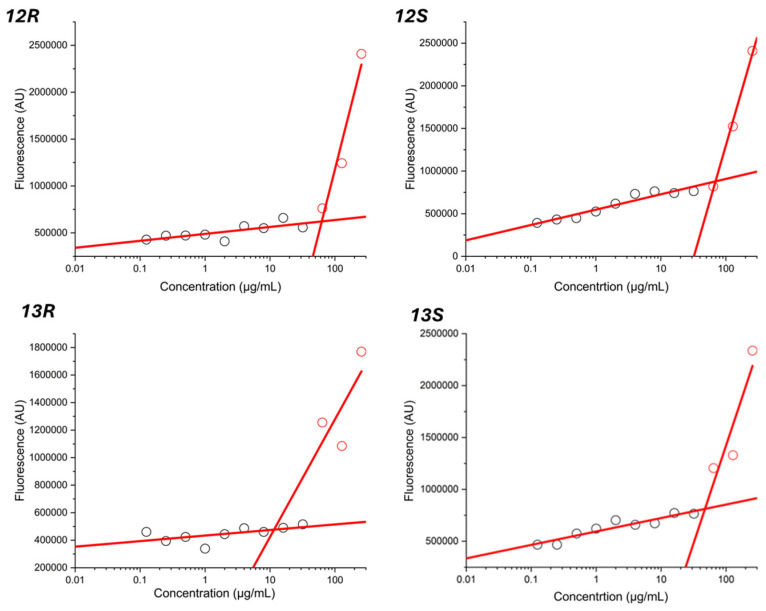
CMC measurements of synthetic humimycin analogs **12*R***, **12*S***, **13*R***, and **13*S***. The CMC is the concentration at the intersection of the linear fits to the fluorescence intensity against log 10 concentration of peptide. Black circle: monomer; red circle: micelle.

The critical micelle concentration (CMC) indicates the concentration at which micelles begin to form in a solution and is an important parameter to study for amphoteric molecules, such as lipopeptides. Below this concentration, the lipopeptide exists primarily as individual molecules (monomers), while above it the molecules aggregate into micelles. The CMC of synthetic humimycin analogs **12*R***, **12*S***, **13*R***, and **13*S*** were determined using the fluorescence intensity as a function of concentration (μg/mL) for each lipopeptide (Figure 4). The CMC values for analogs **12*R***, **12*S***, **13*R***, and **13*S*** were approximately 60, 70, 12, and 40 μg/mL, respectively. In this experiment, Tween-20, a well-known nonionic surfactant with a defined surfactant behavior was used as a positive control to validate the method (Figure S3).

### 2.7. Molecular Docking and Molecular Dynamics Simulation

Molecular docking studies can provide significant insights into the interaction of humimycin analogs with the lipid II flippase MurJ, a crucial protein in bacterial cell wall synthesis [32]. These studies are crucial for understanding the mode of action of humimycins and guide the design and development of new analogs with improved activity and selectivity. The binding of humimycin analogs **12*R***, **12*S***, **13*R***, and **13*S*** to MurJ was first docked using online docking softwares HADDOCK [33] and DockThor [34]. The results showed that all analogs could bind well in the pocket of MurJ, with docking scores ranging from −174 to −193 kcal/mol (Table 4). In addition to the docking results presented in Table 4, molecular docking scores generated using AutoDock Vina are provided to further validate the binding affinity data discussed. A comprehensive analysis of the detailed docking scores is included in Supplementary File (Table S4), offering supplementary insights into molecular interactions.

**Figure 5:**
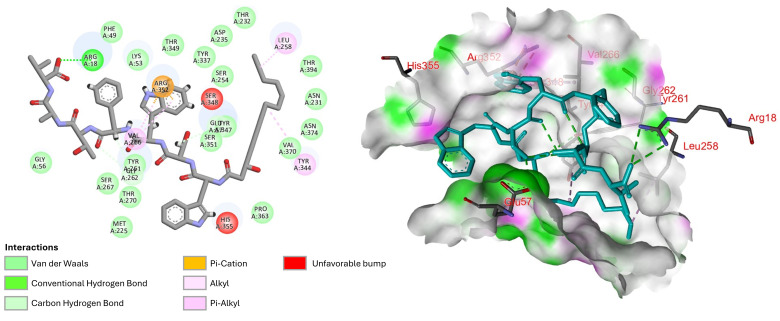
Two-dimensional and three-dimensional illustrations of the interaction of analog **13*S*** with MOP lipid II flippase MurJ [PDB ID:5t77].

Among the tested analogs, peptide **13*S*** exhibited the strongest binding ability, with a total energy of 150.818 kcal/mol, van der Waals interaction energy of −52.443 kcal/mol, and electrostatic interaction energy of −12.309 kcal/mol. These results suggest that hydrophobic interactions play a dominant role in the binding to MurJ. Besides the many van der Waals interactions, docking analysis allowed us to observe an ionic interaction between the analog **13*S*** C-terminal Val7 carboxylate and MurJ Arg18, one pi-cation interaction between d-Trp3 and Arg352, one pi-alkyl interaction between d-Trp3 and Val266, hydrophobic interactions between the lipid chain and Leu258 and Tyr344 side chains (Figure 5).

**Figure 6:**
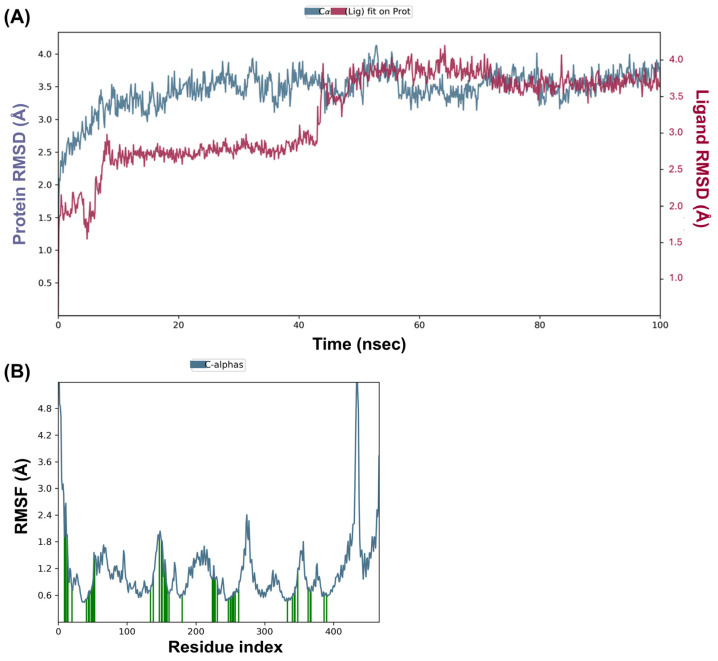
Molecular dynamics (MD) simulation of the MurJ (PDB ID: 5t77) protein and humimycin analog **13*S*** over a 100 ns trajectory. Protein and ligand RMSD (**A**), protein RMSF (**B**).

Molecular dynamics simulations are powerful tools for understanding the dynamic behavior of biomolecular systems at an atomic level. The simulation was conducted with analog **13*S*** on MurJ protein (PDB ID:5t77) using the NPT ensemble at 300 K for 100 ns, involving 54,722 atoms and 15,582 water molecules. The protein RMSD analysis indicated that the system reached equilibrium after approximately 20 ns with fluctuations stabilizing around 2–3 Å, which were within the acceptable range of 1–3 Å, suggesting a stable conformation of the protein throughout the remaining simulation period. On the other hand, the analog **13*S*** RMSD showed that the ligand remained stable within the binding pocket of the protein with RMSD values fluctuating around 1–2 Å after initial equilibration, indicating strong binding affinity and minimal diffusion from the binding site (Figure 6A). RMSF analysis highlighted that the N- and C-terminal regions of the protein exhibited higher fluctuations compared to the core regions, which is typical for protein dynamics. Secondary structure elements such as alpha helices showed lower fluctuations, underscoring their structural rigidity (Figure 6B). In comparison, simulations with apo-MurJ exhibits pronounced conformational flexibility at the C-terminal region, with fluctuations exceeding 5.0 Å (residues 400–450), while the N-terminal domain displays only moderate mobility (~2.0–2.5 Å) (Figure S4). Distinct flexibility peaks are observed at loop regions corresponding to residues ~100, ~200, and ~300, with RMSF values ranging from 2.0 to 3.2 Å (Figure S4B).

## 3. Discussion

A series of humimycin analogs was successfully prepared by solid-phase peptide synthesis [35]. Racemic β-hydroxymyristic acid was used during the synthesis of humimycin analogs **12**–**17**, but the purification of the lipopeptides by HPLC allowed us to obtain the (*S*)- and (*R*)-isomers separately. Among the 19 synthesized analogs, only peptides **12*R***, **12*S***, **13*R***, and **13*S*** exhibited activity against *S. aureus*. These results showed that the humimycin peptide scaffold does not offer a lot of flexibility, as previously reported with the deletion- and alanine-scans [13]. In our case, only modifications at positions 1, 3, and 6, with retention of the amino acids’ stereochemistry, were tolerated. Surprisingly, the substitution of Val at position 6 by Ala led to a four-fold increase in activity against *S. aureus* ATCC 29213, but also to a four-fold decrease in activity against the MRSA strain. The presence of Trp residues at positions 1 and 3 yielded the most active **12*R***, **12*S***, **13*R***, and **13*S***. These substitutions would probably improve the interaction with MurJ and give the optimal amphipathic balance required for membrane interaction and permeabilization. In AMPs, Trp residues often localize at the water-membrane interface, enhancing the peptide’s ability to disrupt bacterial membranes by different interactions. These interactions include hydrogen bonding, hydrophobic contacts, and pi-cation (π-cation) interactions, which help position tryptophan residues at the membrane interface and promote the binding and insertion of the AMPs into the membrane [36]. Additionally, the aromatic side chain of Trp was shown to rapidly form hydrogen bonds with membrane bilayer components [37]. For instance, the synthetic peptide MT-W, which includes Trp residues, exhibited significantly higher antibacterial activity and stability compared to its counterpart without Trp, demonstrating the critical role of Trp in enhancing peptide efficacy and reducing cytotoxicity [37]. In our study, analogs **14*R*** and **14*S*** with a Trp at position 5 instead of Thr may lose the amphipathic balance leading to a loss of activity. On the other hand, it is also possible that the presence of Trp at positions 1 and 3 in analogs **12** and **13** improves binding to MurJ. The results from the molecular docking study support this proposal where a pi-cation interaction between the indole of Trp3 and the guanidinium group of MurJ Arg352 was observed (Figure 5).

C-Terminal amides are commonly found in naturally occurring membrane-disrupting AMPs. If the precise role of C-terminal amidation in the molecular mechanism of action of AMPs is not fully understood, amidation has been shown to simultaneously alters two key properties of a peptide: its net charge and helicity [38]. In our case, substitution of the C-terminal carboxylic acid by an amide group in analogs **15**–**17** also led to a loss of activity. Similar results were observed for the MSI-103 peptide where the strongest antimicrobial activity was obtained with the C-terminal carboxylic acid [39]. These results suggest that the negative charge of the C-terminal carboxylate is essential for activity and could be involved in an ionic interaction with the target. This proposition is supported by the molecular docking study where an ionic interaction between the analog **13*S*** C-terminal Val7 carboxylate and the guanidinium group on the side chain of Arg18 in MurJ was observed (Figure 5).

β-Hydroxymyristic acid is specifically incorporated into lipopeptides like surfactin by certain enzymes in *Bacillus* species [40]. Replacement of the β-hydroxymyristoyl chain with other fatty acids in analogs **5**–**11** resulted in loss of antimicrobial activity. These results suggest that this β-hydroxylated acyl chain is essential for activity with an optimal chain length of 14 carbons, providing suitable hydrophobicity and lipophilicity for interaction with bacterial membranes and binding to MurJ. The presence of a hydroxyl group imparts some amphiphilicity, aiding interaction with bacterial membranes. It has been reported that a moderate chain length (neither too short nor too long) is favorable for disrupting bacterial membrane integrity [41,42]. The substitution of the β-hydroxymyristoyl chain with other fatty acids like geranic acid, dicarboxylic acid, or octanoic acid may disrupt these interactions, modify physicochemical properties, alter binding to the target, and reduce efficacy. For example, geranic acid has an unsaturated carbon chain with a different structure that could alter the hydrophobic interactions of the lipid chain with the bacterial membrane and/or in the binding site of MurJ. In the case of analogs **10** and **11** bearing a dicarboxylic acid, the additional negative charge and increased polarity could disrupt the hydrophobic interactions involved in the mode of action. Finally, with its shorter carbon chain, the octanoyl chain of analog **8** reduces the hydrophobic surface area for interaction and led to a loss of activity. Our study showed that the β-hydroxyl group on the myristoyl chain is important for activity, but its stereochemistry is not. In a previous study, the (*S*)-isomer of humimycin A **1** was found to be four-fold more potent than the (*R*)-isomer [19]. In most cases in our study, equipotency or a two-fold difference in activity was observed between the (***S***)-isomers **12*S*** and **13*S*** and their (*R*)-isomer counterpart **12*R*** and **13*R***. These results suggest that the β-hydroxyl group is probably more involved in the physicochemical properties of the peptides and interaction with the membrane than molecular recognition in the MurJ binding site.

Overall, the structure–activity study unveiled the low flexibility of the humimycin scaffold that only allow very few specific modifications. These results suggest that most residues and functional groups on humimycin and active analogs thereof are involved in the mode of action and specific binding interactions with MurJ. Therefore, unsuitable modifications could lead to inactive analogs by altering the physicochemical and structural properties and weaken or eliminate these critical interactions.

Importantly, analogs **12*R***, **12*S***, **13*R***, and **13*S*** demonstrated rapid inhibitory effects, effectively inhibiting the growth of *S. aureus* at their MIC values shortly after being introduced. Within two hours of exposure, these compounds significantly reduced the bacterial population, indicating interference with essential cellular processes required for bacterial reproduction. The observed speed of kill (swift killing) indicates that these analogs have the potential to be effective therapeutic agents against MDRSA.

Despite their promising antimicrobial activity, analogs **12*R***, **12*S***, **13*R***, and **13*S*** displayed considerable hemolytic activity against human erythrocytes with HC_50_ values ranging from 44.8 to 76.8 µg/mL. Comparing these results with the MIC against *S. aureus* ATCC 29213 gives moderate selectivity index ranging from 3 to 27. However, the selectivity decreased considerably against less sensitive *S. aureus* strains. Factors such as amino acid composition, lipid chain length, net charge, and hydrophobicity play significant roles in determining the hemolytic behavior of antimicrobial lipopeptides. For example, it has been reported that peptides with higher hydrophobicity and a net positive charge show increased antimicrobial activity but also higher hemolytic effect [43]. The hemolytic activity observed for the studied analogs is hypothesized to be due to their high level of hydrophobicity. This observation aligns with findings from lipopeptides like brevibacillin [44,45] and BP100 [46] and their analogs, where hydrophobicity often correlates with hemolytic activity.

The relationship between CMC and MIC values provides insights into the dual functionality of lipopeptides as both antimicrobial agents and surfactants. Evaluation of the CMC for **12*R***, **12*S***, **13*R***, and **13*S*** showed that these peptides most probably interact with the bacterial membrane as monomers because their CMC values were greater than their MIC values. These results are consistent with the observations reported for short lipopeptides [47]. The CMC value is influenced by the hydrophobicity of a lipopeptide, with more hydrophobic molecules generally having lower CMC values. Surprisingly, lower CMC values were observed for analogs **13*R*** and **13*S*** containing a less hydrophobic Ala residue at position 6 instead of a Val for peptides **12*R*** and **12*S***. Stereochemistry of the hydroxyl group was also found to influence CMC as (*R*)-isomers **12*R*** and **13*R*** exhibited lower CMC values than their (*S*)-isomer counterparts **12*S*** and **13*S***. Taken together, these results suggest that micelle formation can also be influenced by the overall structure of the lipopeptide. Generally, lipopeptides with lower MIC also tend to have lower CMC values, indicating that they are effective at lower concentrations and can form micelles more readily. This relationship is significant because it suggests that the antimicrobial activity of lipopeptides is not solely dependent on their ability to disrupt membranes but also on their ability to self-assemble and interact with microbial membranes at sub-micellar concentrations. Further structural and mechanistic studies are warranted to elucidate the underlying reasons for these differences.

Molecular docking and molecular dynamics simulations have become indispensable tools in the field of drug discovery, providing detailed insights into the interactions between small molecules and their target proteins. The first results from the molecular docking study showed that all four humimycin analogs **12*R***, **12*S***, **13*R***, and **13*S*** can bind effectively to the MurJ pocket, with docking scores ranging from −174 to −193 kcal/mol. Among the studied analogs, peptide **13*S*** exhibited the most robust binding affinity, as indicated by its highest total energy score of 150.818 kcal/mol, with significant contributions from van der Waals and electrostatic interactions. The docking data suggest that hydrophobic interactions are the primary forces driving the binding of these analogs to MurJ, which is consistent with the previous findings on the importance of hydrophobic contacts in ligand–receptor interactions [48]. Computational predictions from molecular docking and 100 ns simulations with MurJ offered encouraging preliminary data on the mechanism of action of these lipopeptides. Validating these findings through experimental studies will strengthen the case for their use as effective antimicrobials. Understanding apo-protein RMSD and RMSF provides valuable insights for future studies on the development of antimicrobials targeting MurJ. More especially, the stabilized regions likely represent critical binding hotspots where antimicrobial compounds can more effectively modulate MurJ function. Furthermore, the preservation of the high flexibility in the C-terminal domain (residues 400–450) across both states suggests that ideal inhibitors should induce targeted rigidification at functional sites while allowing certain dynamic regions to maintain their natural mobility, a principle that could guide the rational design of next-generation MurJ inhibitors with enhanced specificity and reduced resistance potential. By elucidating how ligands like humimycin stabilize MurJ, researchers can design more effective inhibitors that exploit these interactions to disrupt bacterial cell wall synthesis, offering new avenues for combating antibiotic resistance.

Overall, this study provides crucial insights on the structure–activity relationship of the humimycin scaffold and paves the way for the design and development of more efficient and safer analogs. The observed, relatively narrow selectivity index offers a promising starting point for optimizing the balance between efficacy and safety. Future work can focus on retuning these analogs to enhance their selectivity index. Additional studies on the efficacy of these peptides in various environments (salt concentrations, pH, temperature) and stability in the presence of enzymes could further demonstrate the robustness of the humimycin analogs and validated their potential for real-world applications. Moreover, the discovery that a micelle formation is not essential for antibacterial activity opens new avenues for exploring how these peptides behave under various physiological conditions, potentially leading to more versatile antimicrobial agents. Additionally, expanding the research to include a broader range of bacterial strains from diverse sources will provide a more comprehensive understanding of the efficacy of these lipopeptides. While the potential for bacterial resistance development was not addressed in this study, this highlights a valuable area for future investigation. Long-term studies will be essential to understand resistance mechanisms and develop strategies to counteract them, ensuring the sustainability of these treatments. By acknowledging these areas for further research, this study sets a positive trajectory for advancing the development of lipopeptide-based therapies to combat MDR *S. aureus* and other resistant bacterial infections.

## 4. Materials and Methods

### 4.1. Solid-Phase Peptide Synthesis, Purification, and Analysis

Peptides **1**, **5**–**17** were prepared by standard solid-phase peptide synthesis (SPPS) using Fmoc/tBu strategy on a Prelude peptide synthesizer (Gyros Protein Technologies, Tucson, AZ) [49]. *N*-Fmoc-protected amino acids, resins, and 2-(7-aza-1H-benzotriazol-1-yl)-1,1,3,3-tetramethyluronium hexafluorophosphate (HATU) were obtained from Matrix Innovations (Quebec, QC, Canada). All other reagents and solvents were acquired from other commercial suppliers at the highest purity grade available for laboratory use and used without further purification. Peptides **1**, **5**–**14** were assembled on Val preloaded 2-chlorotrityl chloride resin (0.75 mmol/g) and analogs **15**–**17** on Rink amide resin (0.65 mmol/g). Briefly, the Fmoc protecting group was removed from the resin by treatments with 20% piperidine in DMF (*v*/*v*) (2 × 7 min) and amino acid couplings were performed with Fmoc-Xaa-OH (3 equiv), HATU (3 equiv) and NMM (6 equiv) in DMF (2 × 20 min). The resin was washed with DMF (5 × 30 s) between every deprotection and coupling steps. After completion of the sequences, the suitable fatty acid was coupled to the N-terminal and the peptides were cleaved from the resin using a trifluoroacetic acid (TFA) cleavage cocktail containing triisopropylsilane (TIS) and water (95:2.5:2.5) for 1.5 h. The resulting peptide was precipitated in cold diethyl ether and hexanes mixture (1:1), and the solid washed twice with the same mixture before drying under a vacuum overnight. The peptides were purified by semi-preparative RP-HPLC with a Shimadzu Prominence system on a Phenomenex Kinetex EVO C18 column (250 × 21.2 mm, 300 Å, 5 µm) using H_2_O (0.1% TFA) (A) and CH_3_CN (0.1% TFA) (B), with a linear gradient of 25–75% for 20 min at a rate of 12 mL/min and detection at 220 and 254 nm. The collected fractions were lyophilized to obtain the desired peptide as a powder. Peptide purity and composition were confirmed by HPLC and mass spectrometry on a Shimadzu Prominence LCMS-2020 system equipped with an electrospray ionization (ESI) probe using a Phenomenex Kinetex EVO C18 column (100 mm × 4.6 mm, 100 Å, 2.6 µm) with a 10.5 min gradient from water (0.1% HCOOH) and CH_3_CN (0.1% HCOOH) (10 to 100% CH_3_CN) and detection at 220 and 254 nm (Table S1, Figure S1). The peptides, obtained as a white powder, were stored at −20 °C.

### 4.2. Antimicrobial Activity Assays

#### 4.2.1. Bacterial Strains and Culture Medias

The bacterial strains used in this study included *S. aureus* ATCC 29213 and ATCC 43300, *B. subtilis* ATCC 23857, *E. coli* ATCC 25922, *P. aeruginosa* ATCC 27853, and *S. enterica* serovar Typhimurium ATCC 14028, all of which were purchased from Cedarlane, Canada. Additionally, a collection of 19 S. aureus strains, ranging from antibiotic-sensitive to multidrug-resistant, and originating from clinical intramammary infections (IMI), was selected from the Mastitis Pathogen Culture Collection of the Canadian Bovine Mastitis Research Network (Université de Montréal, St-Hyacinthe, QC, Canada) [50]. These strains, previously used in other studies [30], were also included in this work. *S. aureus* ATCC 29213 and *B. subtilis* ATCC 6633 were cultured in Tryptic Soy Broth (TSB) (Difco^TM^, Becton, Dickenson & Company, Spark, MD, USA). *E. coli* ATCC 25922 and *P. aeruginosa* ATCC 27853 were grown in Luria–Bertani (LB) broth (Fisher Scientific, Fair Lawn, NJ, USA). *S. enterica* serovar Typhimurium ATCC 14028 was propagated in Nutrient Broth (NB) (Oxoid, Basingstoke, UK). All bacterial cultures were incubated overnight at 37 °C before use and during the performance of assays.

#### 4.2.2. Minimal Inhibitory Concentration and Agar Diffusion Assays

MIC assays were performed using the broth microdilution method in 96-well plates [51]. Analogs were serially diluted two-fold from stock solutions. The plates were incubated overnight at 37 °C and bacterial growth was monitored by measuring the absorbance at 595 nm over 18–20 h and the assay was performed in triplicate. The MIC was defined as the lowest concentration of the analog that resulted in complete inhibition of bacterial growth.

For the agar diffusion assay, the bacterial strain was cultured overnight at 37 °C in the previously described media. Cultures were then inoculated at 1% (*v*/*v*) in a soft agar medium containing 1.5% (*w*/*v*) agar. Wells were created in the inoculated agar plates, and 70 µL of the humimycin analog solution (256 µg/mL) was added to each well. Kanamycin sulfate (256 µg/mL) and 5% dimethyl sulfoxide (DMSO) in water were included as positive and negative control, respectively.

### 4.3. Bacterial Growth Experiments

The growth kinetics of *S. aureus* ATCC 29213 in presence of humimycin analogs **12*R***, **12*S***, **13*R***, and **13*S*** was assessed on *S. aureus* cultures in TSB at 37 °C in the stationary phase. The overnight culture was then diluted 1:100 in fresh TSB to achieve an initial optical density at 600 nm (OD600) of approximately 0.05. Humimycin analogs were prepared at concentrations corresponding to 0.5×, 1×, and 2× the MIC. *S. aureus* cultures without any analog served as the positive control and culture media was the negative control. The treated and control cultures were incubated at 37 °C for a total duration of 6 h. Absorbance readings at 595 nm were recorded every 2 h using a microplate reader to monitor the bacterial growth. The OD_595_ values were then plotted against time to generate bacterial growth curves. To ensure reproducibility, all experiments were performed in triplicate, with results expressed in a graph as mean ± standard deviation.

### 4.4. Hemolytic Activity Assay

Hemolytic potential of the humimycin analogs **12*R***, **12*S***, **13*R***, and **13*S***, was evaluated by exposing fresh, uninfected red blood cells (RBCs) to 16 two-fold serial dilutions of each analog, ranging from 512 μg/mL to 0.015 μg/mL, in RPMI-1640 medium supplemented with HEPES and containing a maximum of 0.5% dimethyl sulfoxide (DMSO). The assay was performed in round-bottom 96-well plates at a final hematocrit of 1%. Mock control wells contained RBCs in RPMI-1640 with 1% DMSO, while 100% lysis control wells contained RBCs in RPMI-1640 with 1% DMSO and 1% Triton X-100. The plates were incubated for 1 h at 37 °C under a gassed atmosphere. Following incubation, the plates were centrifuged at 800× *g* for 5 min, and 100 μL of supernatant from each well was transferred to a clean flat-bottom 96-well plate. The absorbance of released hemoglobin in the supernatant was measured at 405 nm using a VICTOR plate reader (PerkinElmer). The percentage of hemolysis was calculated by comparing the absorbance of the supernatant from compound-treated RBCs to that of the mock control and 100% lysis control wells. All assays were performed in triplicates.

### 4.5. Critical Micelle Concentration Determination

The CMC of synthesized analogs **12*R***, **12*S***, **13*R***, and **13*S*** was determined using the Nile Red fluorescence method, which takes advantage of the dye’s enhanced fluorescence emission in hydrophobic environments. A 500 μM stock solution of Nile Red was prepared in DMSO. Aliquots of 10 μL of this stock solution were added to each well and kept in the dark. Peptide solutions (200 μL) at various concentrations were then added to the well and allowed to equilibrate with the Nile Red overnight. Fluorescence emission spectra were recorded from 485 nm to 636 nm on a SpectraMax i3 spectrofluorometer with an excitation and emission bandwidth 9 nm and 15 nm, respectively. The ratio of the emission intensity was calculated for each peptide concentration and plotted against the logarithm of the peptide concentration. The CMC was determined from the intersection of the linear fits to the data points below and above the transition region in the plot. Tween-20 was used as positive control.

### 4.6. Molecular Docking Study

An investigation of the interactions between the multidrug/oligosaccharidyl lipid/polysaccharide (MOP) flippase MurJ and analog **13*S*** was performed through a series of computational methods. First, the Protein Data Bank (PDB) was searched for the crystal structure of MOP flippase MurJ, identified by PDB ID: 5t77. Next, the protein structure for docking was prepared using AutoDock MGL Tools v. 1.5.6. This involved opening the downloaded PDB file and removing any water molecules, heteroatoms, and other non-protein components. Necessary corrections such as adding missing hydrogen atoms and assigning charges were performed. To optimize the protein structure, energy minimization and refinement techniques were applied using appropriate force fields and optimization algorithms. The structure of peptide ligand **13*S*** was generated using ChemDraw professional 21.0 and imported into AutoDock MGL Tools. Charges and atom types were assigned to the ligand and its structure converted to a compatible format for further analysis using AutoDock Vina 1.1.2 [52]. Computational tools like CASTp 3.0 [53] were employed to identify potential binding sites or protein pockets within MurJ. The binding sites predicted by CASTp 3.0 during modeling were chosen for molecular docking studies (Table S2). The molecular docking of peptide **13*S*** into the selected protein pockets was performed using AutoDock Vina. The docking parameters were set, including the grid box dimensions (42, 40, and 38 for X, Y, Z coordinates of the protein), energy range (4), and exhaustiveness (8). The space around the protein pockets was defined followed by configuration of the scoring functions and search algorithms (Table S3). The software conducted the docking simulation, exploring ligand conformations and orientations to identify the most favorable binding poses based on scoring and interaction analysis. Finally, the protein-ligand interactions were visualized using PyMOL v2.5.4 [54] and BioVia Discovery Studio Visualizer v21.1.0.20298 [55]. The protein-ligand complex obtained from the molecular docking was loaded into these tools, allowing visualization of key interactions.

### 4.7. Molecular Dynamics Simulation

To investigate the dynamic behavior and stability of MurJ (5t77) and compound **13*S*** complexes, MD simulations were performed using the Desmond software package. Initially, the protein-ligand complexes obtained from the docking step were prepared for MD simulations. The structures were solvated in an explicit water box, ensuring that the entire complex was surrounded by water molecules to mimic the physiological environment. Counterions were added to neutralize the overall charge of the system, followed by energy minimization to remove any unfavorable contacts and relax the initial structure. Following energy minimization, the system underwent a stepwise equilibration process to reach a stable state. This equilibration was performed in two stages: NVT (constant number of particles, volume, and temperature) and NPT (constant number of particles, pressure, and temperature) ensembles. During the NVT equilibration, the system temperature was gradually increased from an initial value to the desired simulation temperature, with harmonic restraints applied to the protein and ligand atoms to maintain their overall structure while allowing the solvent and ions to adjust. Subsequently, during the NPT equilibration, the system pressure was gradually equilibrated. The position restraints on the protein and ligand were further reduced to allow greater flexibility in the system. The pressure was maintained at the desired value using the Martyna-Tobias-Klein barostat, which adjusted the system volume to maintain the set pressure. Once equilibrated, MD simulations were initiated and conducted for a total duration of 100 ns to capture sufficient dynamics of the protein-ligand interactions. The simulations were performed under periodic boundary conditions to simulate an infinite system, allowing molecules to move freely. The OPLS-AA force field was employed to describe the protein and ligand parameters during the simulations. The equations of motion were solved using the Verlet integrator with a time step of 2 fs. Throughout the MD simulations, the coordinates and energies of the system were recorded at regular intervals. The resulting trajectories were then analyzed to gain insights into various aspects of the protein-ligand complexes. This methodology ensures a comprehensive approach to studying the dynamic behavior and stability of the protein-ligand complexes, providing valuable insights into their interactions and potential functional implications.

## 5. Conclusions

Our study highlights the potential of the lipopeptide humimycin and its analogs as effective antimicrobials against multidrug-resistant *S. aureus*. Exploration of the humimycin scaffold via a structure–activity study showed that the β-hydroxymyristoyl chain and C-terminal carboxylic acid are essential for antimicrobial efficacy, and that only very few specific modifications are allowed before a complete loss of activity. Despite these constraints, our study identified four humimycin analogs with significant antibacterial activity against a wide range of antibiotic-sensitive and antibiotic-resistant *S. aureus*. Given the critical need for alternative strategies to combat MDRSA and other drug-resistant pathogens, our research endeavors to contribute to the global fight against antibiotic resistance, offering hope for the advancement of new therapies that can safeguard public health.

## Figures and Tables

**Table 2:** The MIC (µg/mL) of humimycin A and its analogs against Gram-positive and Gram-negative bacteria.

Pathogens	Peptide				
	1	12*R*	12*S*	13*R*	13*S*
*Staphylococcus aureus* ATCC 29213	8	16	8	4	2
*Staphylococcus aureus* ATCC 43300 ^a^	16	16	16	128	64
*Bacillus subtilis* ATCC 23857	- ^b^	-	-	-	-
*Escherichia coli* ATCC 25922	-	-	-	-	-
*Pseudomonas aeruginosa* ATCC 27853	-	-	-	-	-
*Salmonella enterica* ATCC 14028	-	-	-	-	-

^a^ Methicillin-oxacillin resistant *S. aureus*. ^b^ No antimicrobial activity was detected up to 256 µg/mL.

**Table 3:** MIC values (µg/mL) of humimycin analogs against the collection of *S. aureus* isolates.

*S. aureus* Strain	Antibiotic Resistance Profile ^a^	12*R*	12*S*	13*R*	13*S*
4-121		32	32	256	256
4-131		>256	>256	>256	>256
4-133	PEN-CEF-CTX-FOX-CIP-CHL-KAN-GEN	256	256	>256	>256
4-134	PEN-VAN-CEF-CTX-FOX-ERY-CC-TET-KAN-GEN-STR	>256	>256	>256	>256
4-138		4	4	16	16
4-139	PEN-ERY-CC-TET-KAN	4	8	8	8
4-140	TET	8	16	16	16
4-144	PEN-AMC-CEF-CTX-FOX-CIP-CHL-ERY-TET	8	16	8	16
4-145		16	32	16	32
4-153		16	16	32	32
4-156		4	8	16	8
4-157		16	16	32	32
4-159		8	16	16	8
4-162		>256	>256	>256	>256
A-165	PEN-CEF-CTX-FOX-CIP-CC-KAN-GEN-STR-P/N	256	256	>256	>256
4-170		4	8	8	8
4-171	PEN-AMC-CEF-CTX-FOX	2	1	0.5	8
4-172	PEN-AMC-CEF-CTX-FOX	8	8	8	8
4-173		8	16	8	8

^a^ Resistance profile of the *S. aureus* strains described as their ability to withstand various antibiotics, represented by three-letter abbreviations corresponding to the following: PEN, penicillin; CEF, cephalothin; CTX, cefotaxime; FOX, cefoxitin; CIP, ciprofloxacin; CHL, chloramphenicol; KAN, kanamycin; GEN, gentamicin; VAN, vancomycin; ERY, erythromycin; CC, clindamycin; TET, tetracycline; STR, streptomycin; AMC, amoxicillin/clavulanic acid; P/N, penicillin/novobiocin).

**Table 4:** Molecular docking results of humimycin analogs.

Analog	Docking Score by Hepdoc	DockThor			
		Affinity	Total Energy (kcal/mol)	van der Waals Energy (kcal/mol)	Electrostatic Energy (kcal/mol)
**12*R***	−193.350	−11.035	139.473	−38.426	−34.328
**12*S***	−174.120	−10.287	144.212	−33.921	−35.708
**13*R***	−187.006	−11.170	150.169	−37.841	−18.467
**13*S***	−191.760	−10.618	150.818	−52.443	−12.309

## Data Availability

The original contributions presented in this study are included in the article/Supplementary Material. Further inquiries can be directed to the corresponding author.

## Supplementary Materials

The following supporting information can be downloaded at: https://www.mdpi.com/article/10.3390/antibiotics14040385/s1. Table S1: Chemical characterization of synthesized peptides; Table S2: CASTp analysis, including pocket ID, surface area (SA), and volume (SA); Table S3: Key residues associated with each binding pocket of MurJ (PDB ID: 5t77); Table S4: Molecular docking results with Autodock Vina; Figure S1: HPLC profiles (λ = 220 nm) and ESI-MS spectra of synthesized peptides **1**, **5**–**17**; Figure S2: Agar diffusion assay of synthesized peptides **1**, **5**–**11** and **14**–**17** against *S. aureus* ATCC 29213; Figure S3: Reference control: Tween-20; Figure S4: Molecular dynamics (MD) simulation of the Apo-MurJ (PDB ID: 5t77) protein over a 100 ns trajectory.
