# Supplementary material for: Structure–Activity Study of the Antimicrobial Lipopeptide Humimycin A and Screening Against Multidrug-Resistant Staphylococcus aureus
Source: Antibiotics (Basel). 2025 Apr 5;14(4):385. doi: 10.3390/antibiotics14040385 (PMC12024397; doi:10.3390/antibiotics14040385)
Supplement: Supplementary file 1 [file antibiotics-14-00385-s001.zip › antibiotics-3535034-supplementary.pdf]

Supporting Information

Structure-Activity Study of the Antimicrobial Lipopeptide Humimycin A and Screening Against Multidrug-Resistant *Staphylococcus aureus*

Md Ramim Tanver Rahman[1,2,3,4], Louis-David Guay[1,2,3,4], Ismail Fliss[3,4,5] and Eric Biron[1,2,3,4,*]

1 Faculty of Pharmacy, Université Laval, Québec, Québec G1V 0A6, Canada;
2 Laboratory of Medicinal Chemistry, CHU de Québec-Université Laval Research Center, Québec, Québec G1V 4G2, Canada
3 Institute of Nutrition and Functional Foods, Université Laval, Québec, Québec G1V 0A6, Canada
4 Research Center in Infectious Diseases, Université Laval, Québec, Québec G1V 0A6, Canada
5 Department of Food Science, Faculty of Agriculture and Food Sciences, Université Laval, Québec, Québec G1V, Canada

* Correspondence: eric.biron@pha.ulaval.ca; Tel. +1-418-524-2296

Table of Contents

Table S1. Chemical characterization of synthesized peptides. S2

Figure S1. HPLC profiles (λ = 220 nm) and ESI-MS spectra of synthesized peptides **1**, **3**, **5-17**. S3

Figure S2. Agar diffusion assay of synthesized peptides **5-11** and **14-17** against *S. aureus* ATCC 29213 .... S18

Figure S3. Reference control: Tween-20. The CMC is the concentration at the intersection of the linear fits to the fluorescence intensity against concentration. S19

Figure S4. Molecular dynamics (MD) simulation of the Apo-MurJ (PDB ID: 5t77) protein over a 100 ns trajectory. Apo-Protein RMSD (A), Apo-Protein RMSF (B), Apo-Protein secondary structure (C). S20

Table S2. CASTp analysis, including pocket ID, surface area (SA), and volume (SA) S21

Table S3. Key residues associated with each binding pocket of MurJ (PDB ID: 5t77) S22

Table S4. Molecular docking results with Autodock Vina. S24

Table S1. Chemical characterization of synthesized peptides.

#	Peptide	Chemical formula	HPLC rt (min)	Yields (%)	Purity (%)	LRMS (ESI) (da)	
						Calculated	Observed
**1**	Hum A	$C_{58}H_{85}N_7O_{14}$	***R***: 12.20<br>***S***: 12.33	5<br>5	95<br>91	$[M+H]^+$ 1104.62<br>$[M+Na]^+$ 1126.60	1104.55<br>1126.50
**5**	Hum A_isoC15	$C_{59}H_{85}N_7O_{13}$	13.20	3	85	$[M+H]^+$ 1100.63<br>$[M+Na]^+$ 1122.61	1100.45<br>1122.70
**6**	Hum A_isoC13	$C_{57}H_{81}N_7O_{13}$	12.36	5	95	$[M+H]^+$ 1072.60<br>$[M+Na]^+$ 1094.58	1072.50<br>1094.70
**7**	Hum W_geranoyl	$C_{56}H_{74}N_8O_{12}$	12.38	4	95	$[M+H]^+$ 1051.55<br>$[M+Na]^+$ 1073.53	1051.65<br>1073.35
**8**	Hum W_octanoyl	$C_{54}H_{74}N_8O_{12}$	11.57	5	94	$[M+H]^+$ 1027.55<br>$[M+Na]^+$ 1049.53	1027.45<br>1049.40
**9**	Hum W_mandelic	$C_{54}H_{66}N_8O_{13}$	10.57	4	90	$[M+H]^+$ 1035.48<br>$[M+Na]^+$ 1057.46	1035.01<br>1057.51
**10**	Hum W_maleic	$C_{50}H_{62}N_8O_{14}$	13.53	3	87	$[M+H]^+$ 999.45<br>$[M+Na]^+$ 1021.43	999.35<br>1021.25
**11**	Hum W_fumaric	$C_{50}H_{62}N_8O_{14}$	9.95	3	85	$[M+H]^+$ 999.45<br>$[M+Na]^+$ 1021.43	999.45<br>1021.43
**12**	Hum W1,3	$C_{62}H_{87}N_9O_{12}$	***R***: 13.33<br>***S***: 13.58	5<br>5	94<br>95	$[M+H]^+$ 1150.66<br>$[M+Na]^+$ 1172.64	1150.50<br>1172.45
**13**	Hum W1,3-A6	$C_{60}H_{83}N_9O_{12}$	***R***: 13.10<br>***S***: 13.37	5<br>5	91<br>94	$[M+H]^+$ 1122.62<br>$[M+Na]^+$ 1144.61	1122.45<br>1144.51
**14**	Hum W1,3,5	$C_{69}H_{90}N_{10}O_{11}$	***R***: 13.73<br>***S***: 14.16	4<br>5	92<br>95	$[M+H]^+$ 1235.69<br>$[M+Na]^+$ 1257.67	1235.75<br>1257.90
**15**	Hum W amide	$C_{60}H_{87}N_9O_{12}$	***R***: 12.90<br>***S***: 13.20	4<br>5	90<br>92	$[M+H]^+$ 1126.66<br>$[M+Na]^+$ 1148.64	1126.95<br>1148.65
**16**	Hum W1,3 amide	$C_{62}H_{88}N_{10}O_{11}$	***R***: 12.85<br>***S***: 13.02	5<br>4	89<br>92	$[M+H]^+$ 1149.67<br>$[M+Na]^+$ 1171.65	1149.50<br>1171.55
**17**	Hum W1,3-A6 amide	$C_{60}H_{84}N_{10}O_{11}$	***R***: 13.42<br>***S***: 13.59	3<br>5	85<br>91	$[M+H]^+$ 1121.64<br>$[M+Na]^+$ 1143.62	1121.50<br>1143.45

Figure S1. HPLC profiles (λ = 220 nm) and ESI-MS spectra of synthesized peptides **1**, **3**, **5-17**.

Humimycin A (**1**)

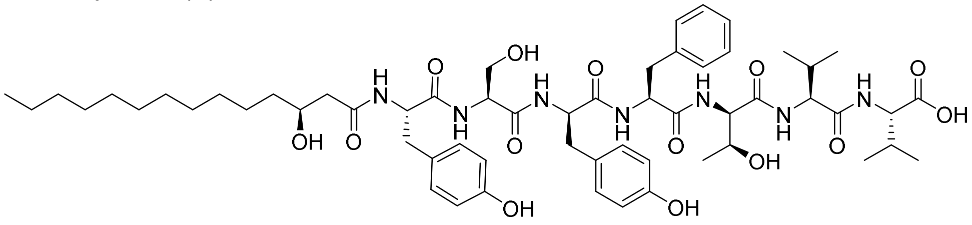

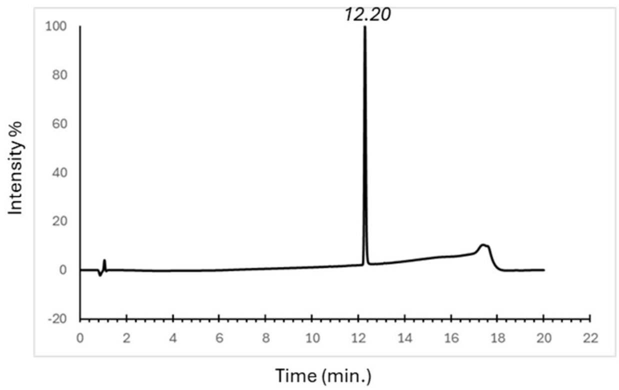

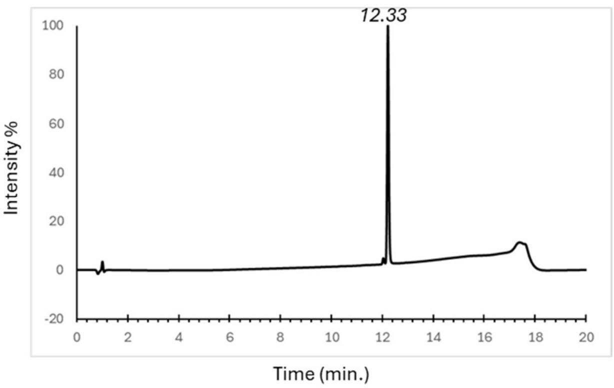

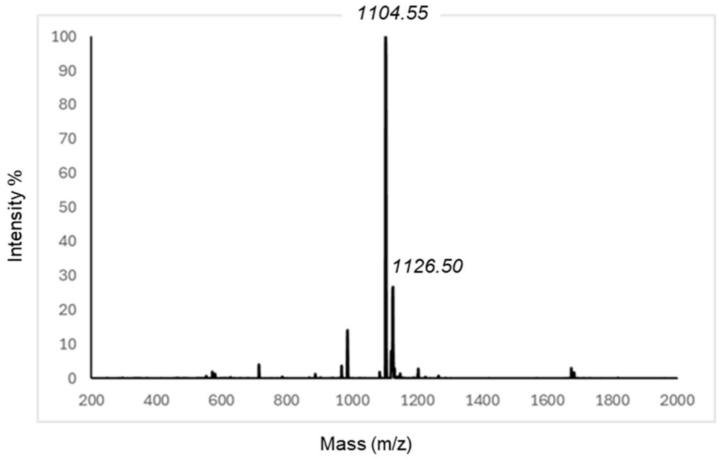

Calculated for $C_{58}H_{85}N_7O_{14}$	
$[M+H]^+$	1104.62
$[M+Na]^+$	1126.60

Figure S1. (Continued)

Humimycin W (**3**)

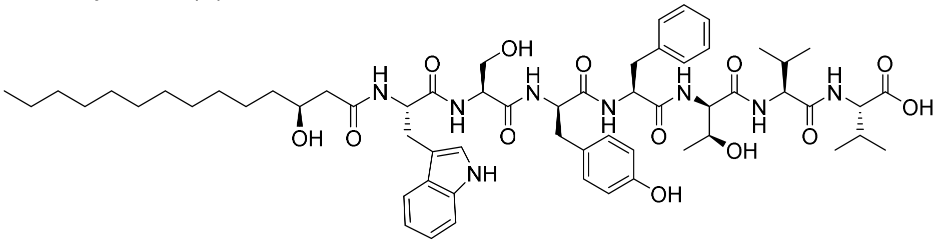

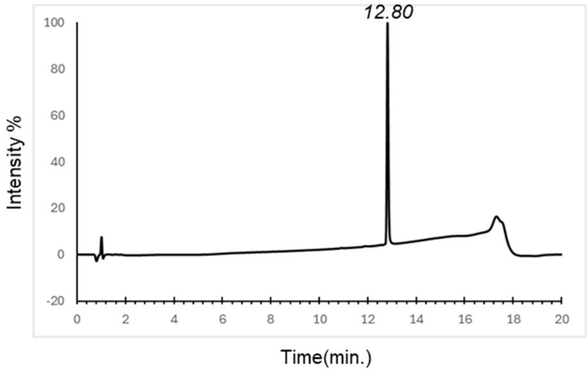

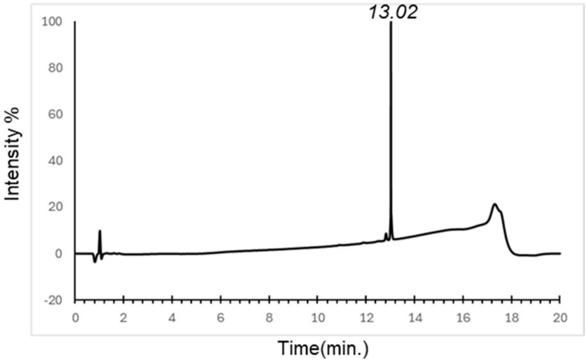

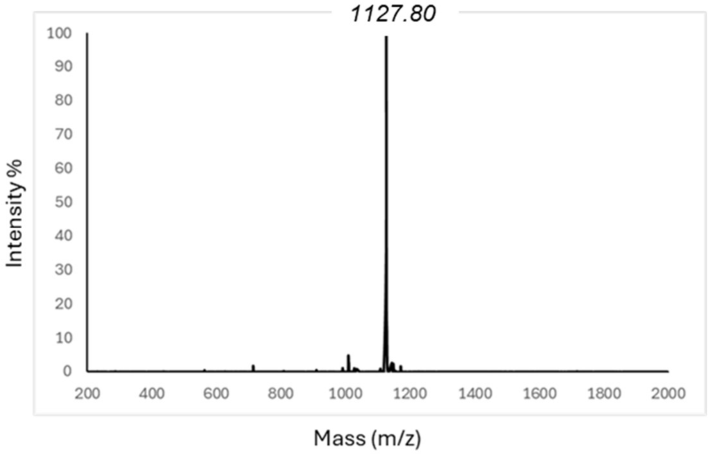

Calculated for $C_{60}H_{86}N_8O_{13}$	
$[M+H]^+$	1127.64

Figure S1. (Continued)

Hum A_isoC15 (**5**)

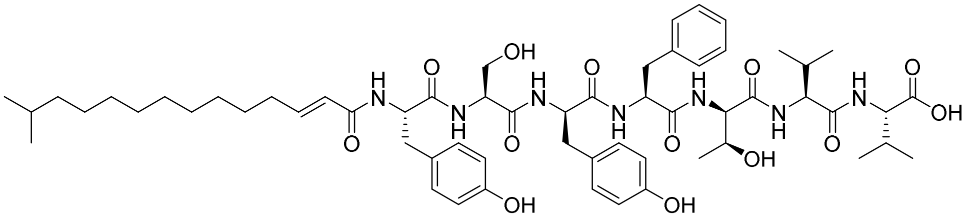

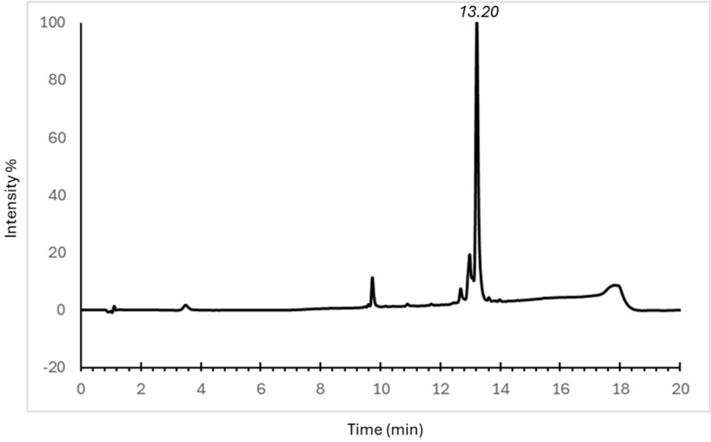

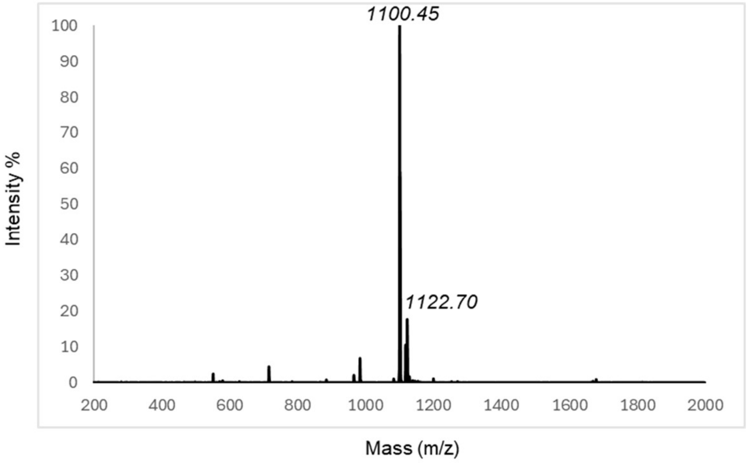

Calculated for $C_{59}H_{85}N_7O_{13}$	
$[M+H]^+$	1100.63
$[M+Na]^+$	1122.61

Figure S1. (Continued)

Hum A_isoC13 (**6**)

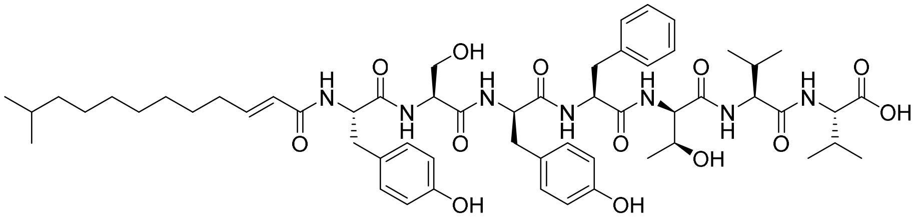

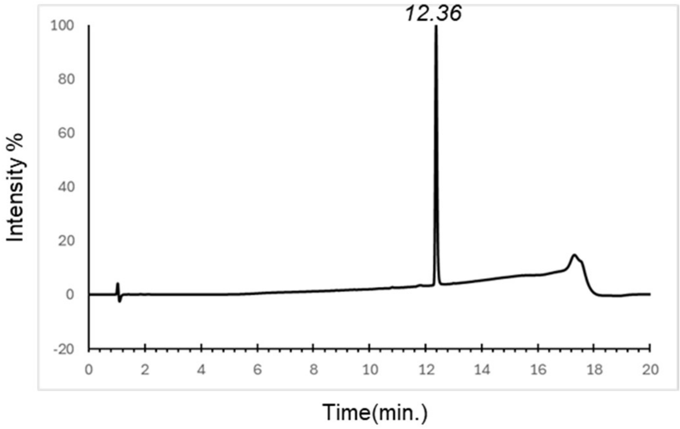

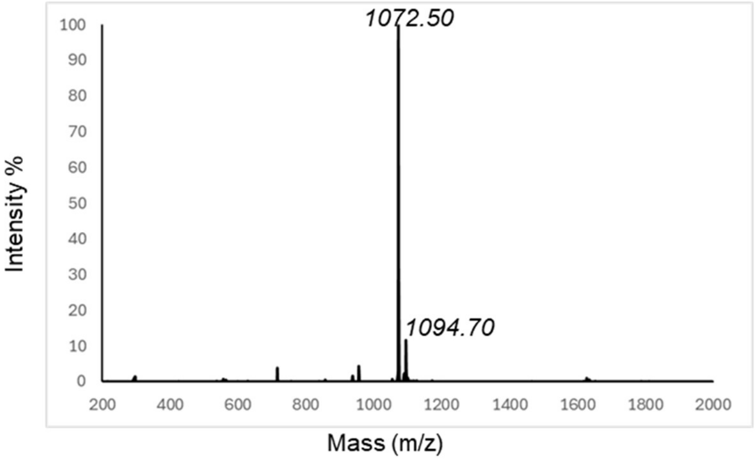

Calculated for $C_{57}H_{81}N_7O_{13}$	
$[M+H]^+$	1072.60
$[M+Na]^+$	1094.58

Figure S1. (Continued)

Hum W_geranoyl (**7**)

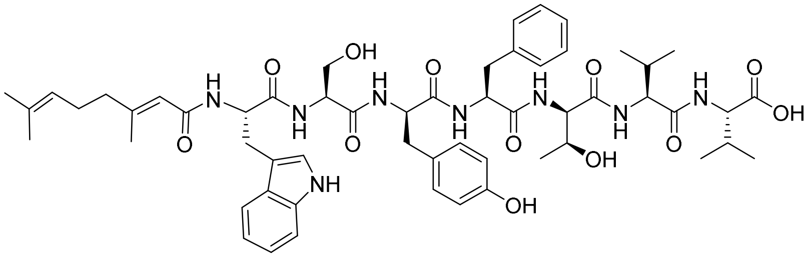

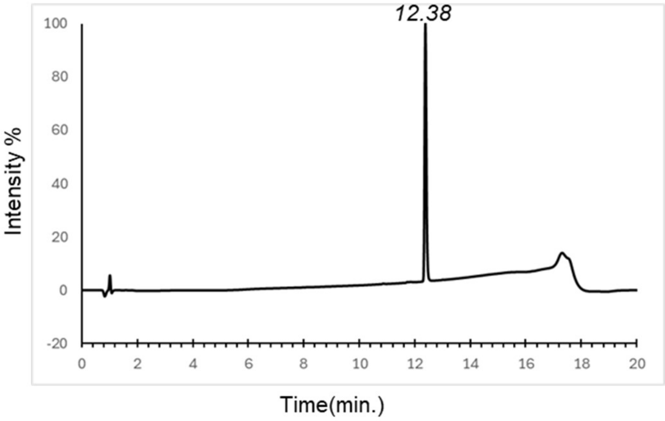

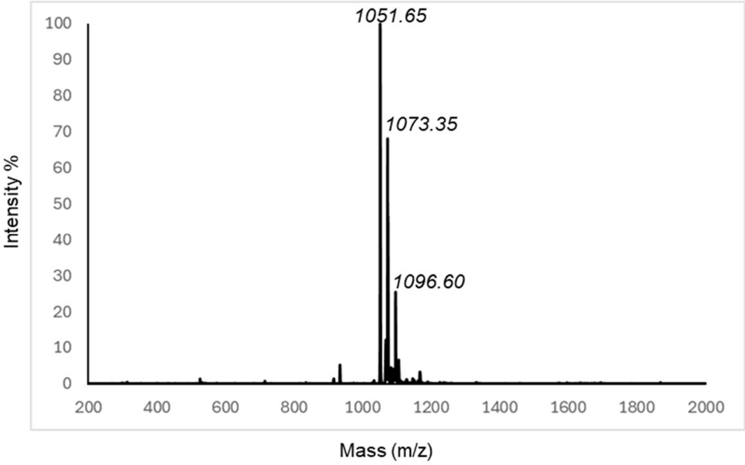

Calculated for $C_{56}H_{74}N_8O_{12}$	
$[M+H]^+$	1051.55
$[M+Na]^+$	1073.53

Figure S1. (Continued)

Hum W_octanoyl (**8**)

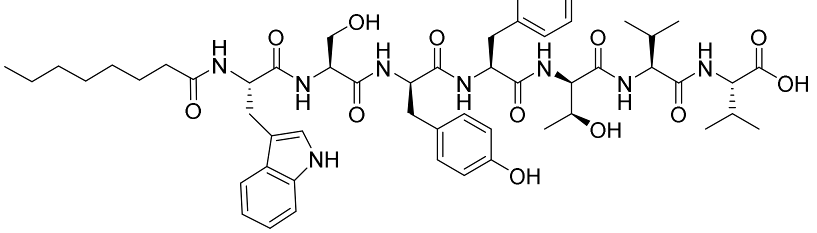

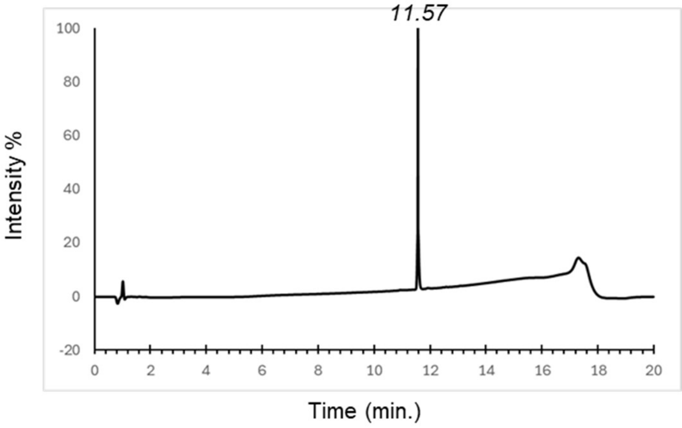

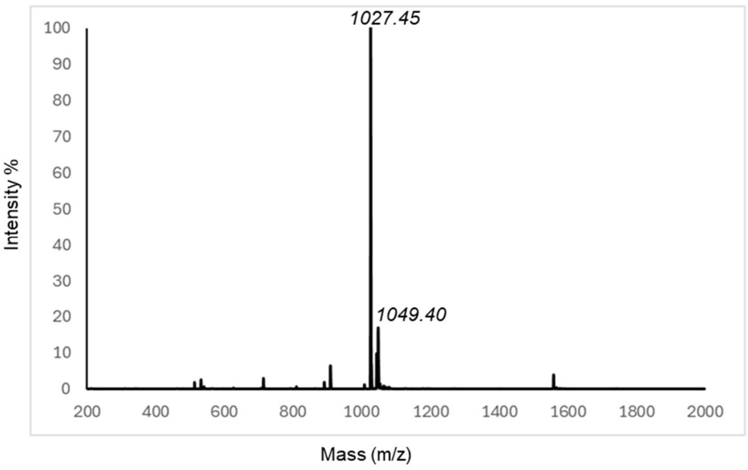

Calculated for $C_{54}H_{74}N_8O_{12}$	
$[M+H]^+$	1027.55
$[M+Na]^+$	1049.53

Figure S1. (Continued)

Hum W_mandelic (**9**)

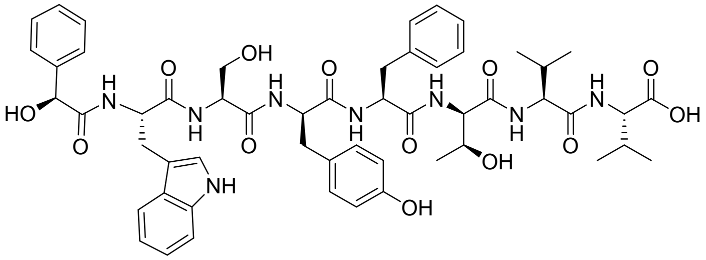

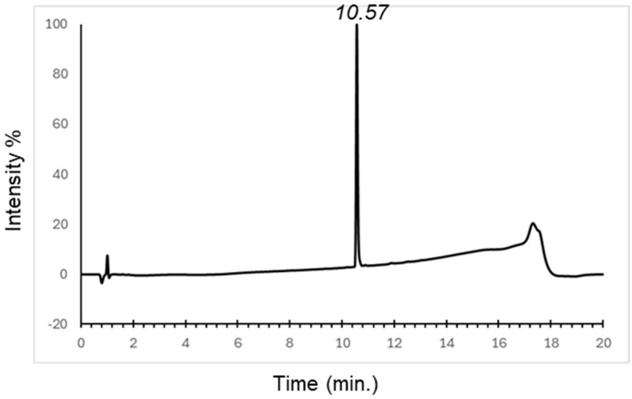

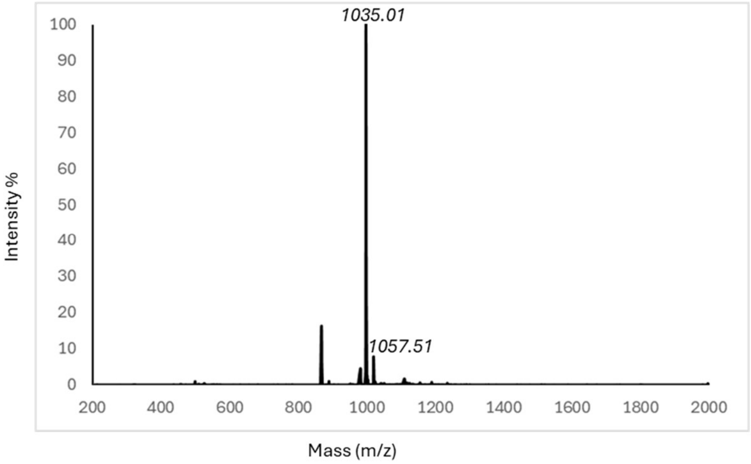

Calculated for $C_{54}H_{66}N_8O_{13}$	
$[M+H]^+$	1035.48
$[M+Na]^+$	1057.46

Figure S1. (Continued)

Hum W_maleic (**10**)

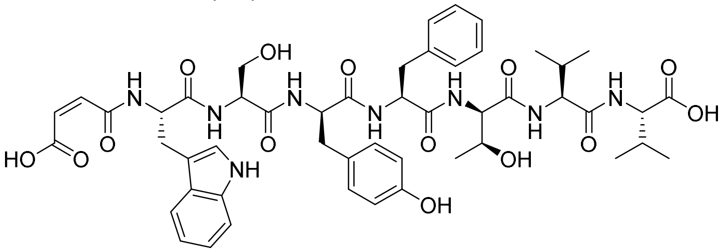

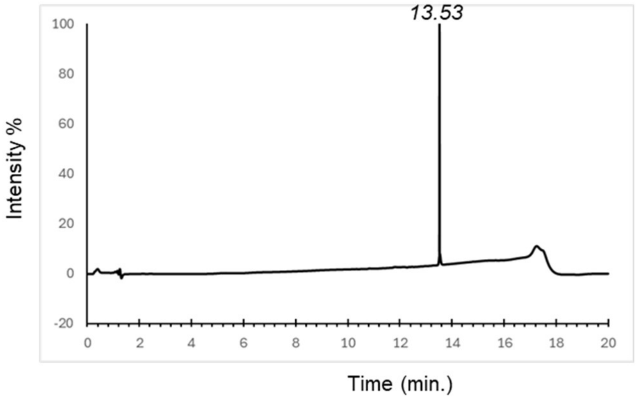

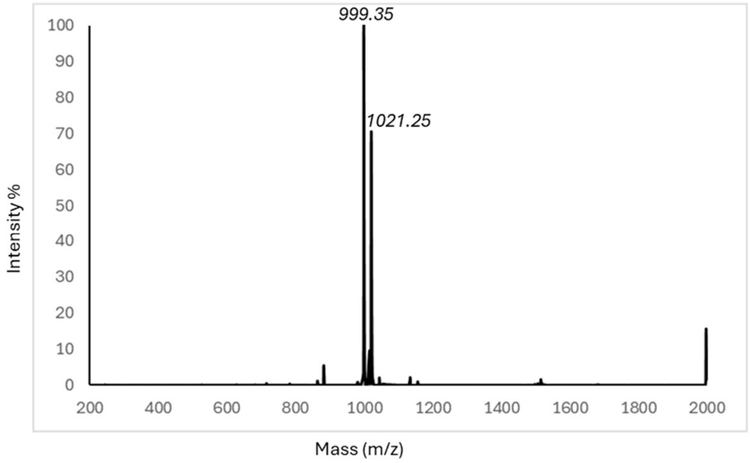

Calculated for $C_{50}H_{62}N_8O_{14}$	
$[M+H]^+$	999.45
$[M+Na]^+$	1021.43

Figure S1. (Continued)

Hum W_fumaric (**11**)

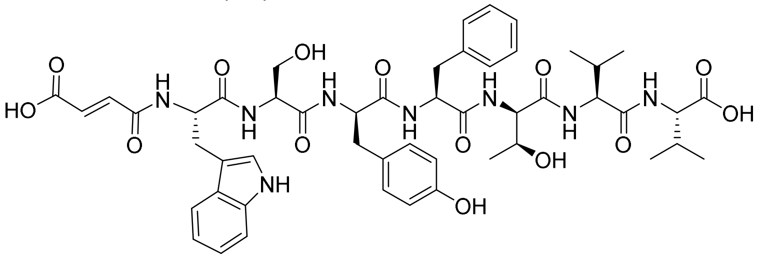

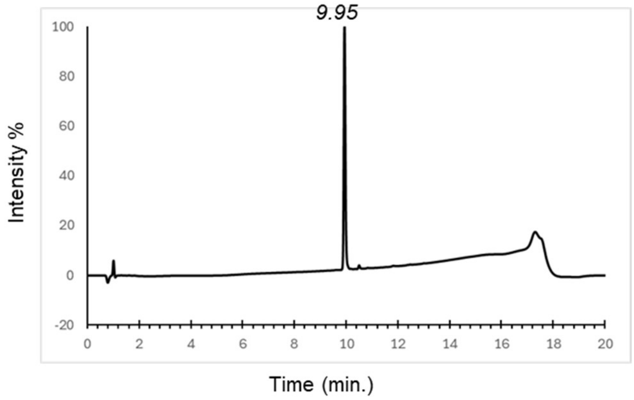

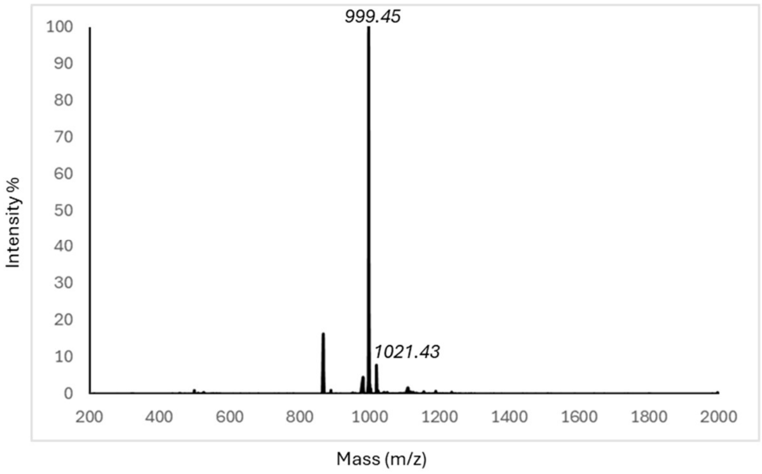

Calculated for $C_{50}H_{62}N_8O_{14}$	
$[M+H]^+$	999.45
$[M+Na]^+$	1021.43

Figure S1. (Continued)

Hum W1,3 (**12**)

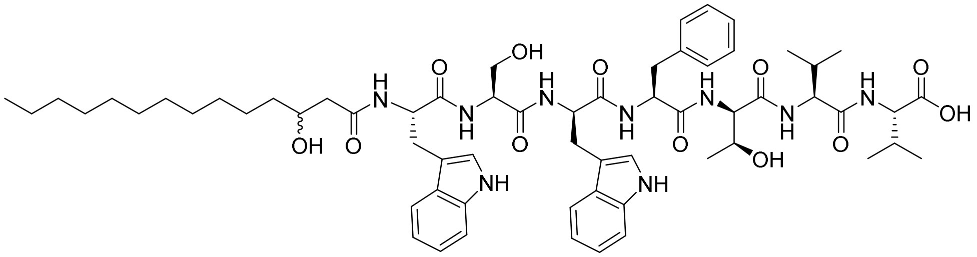

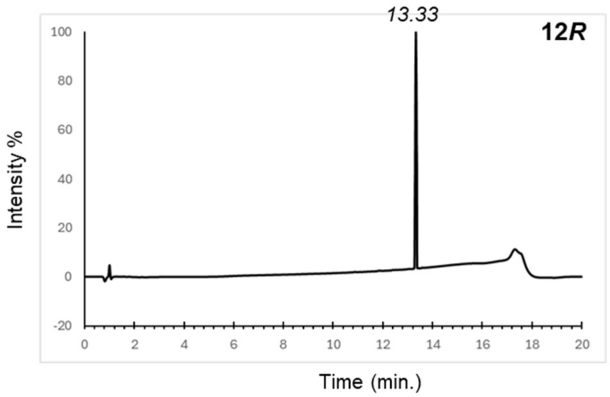

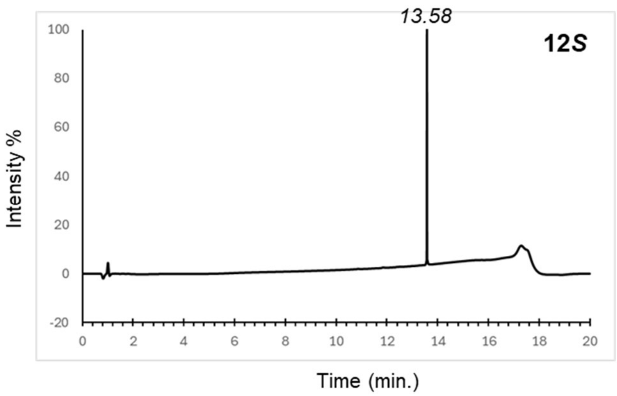

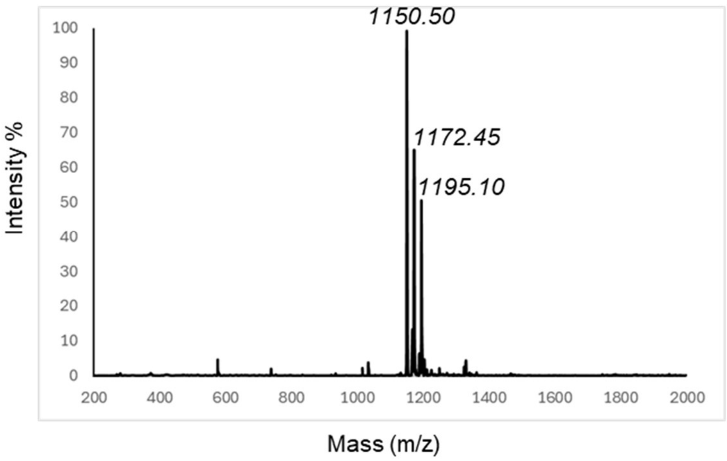

Calculated for $C_{62}H_{87}N_9O_{12}$	
$[M+H]^+$	1150.66
$[M+Na]^+$	1172.64

Figure S1. (Continued)

Hum W1,3-A6 (**13**)

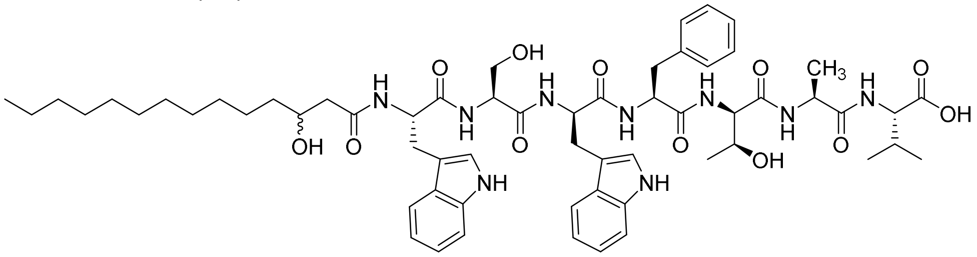

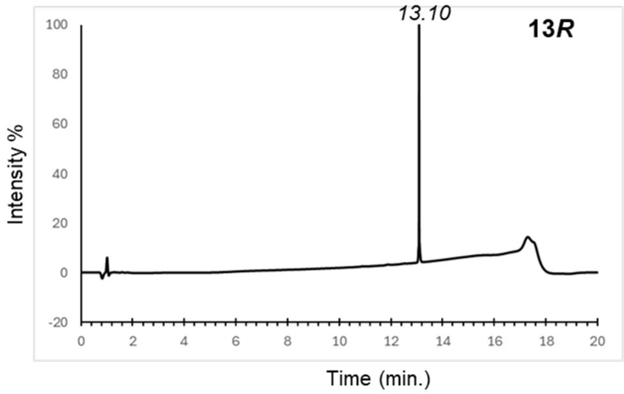

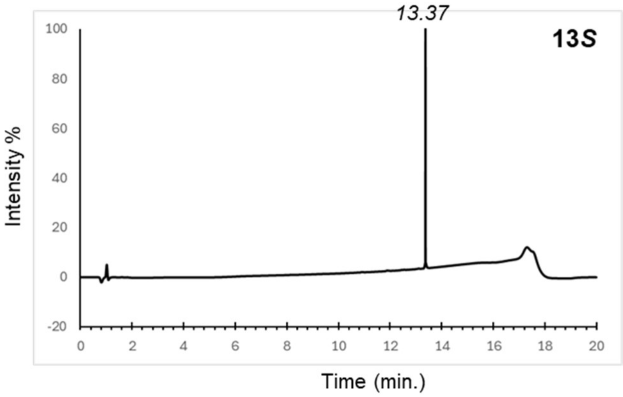

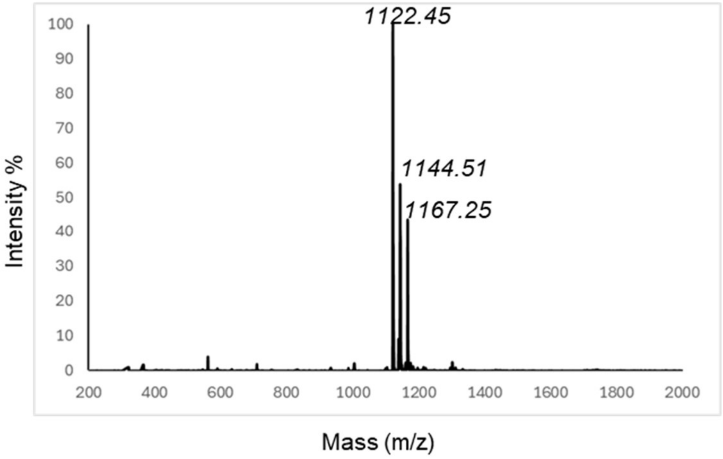

Calculated for $C_{60}H_{83}N_9O_{12}$	
$[M+H]^+$	1122.62
$[M+Na]^+$	1144.61

Figure S1. (Continued)

Hum W1,3,5 (**14**)

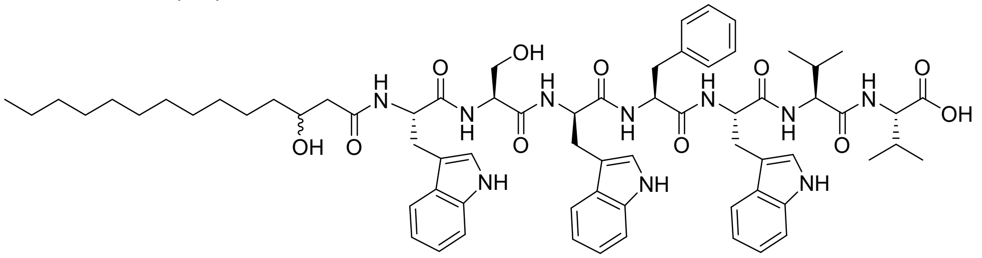

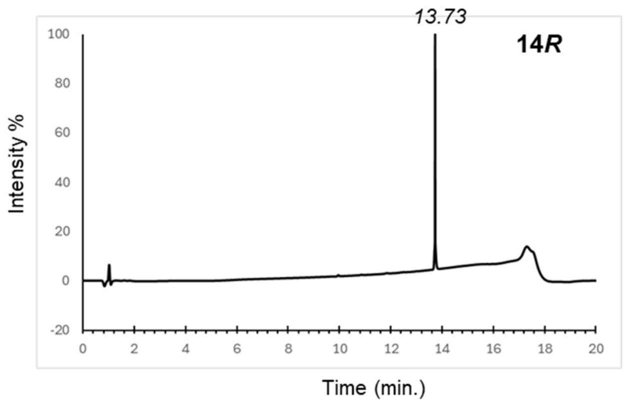

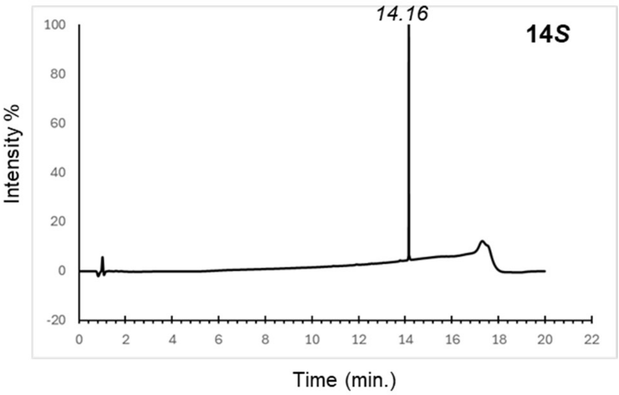

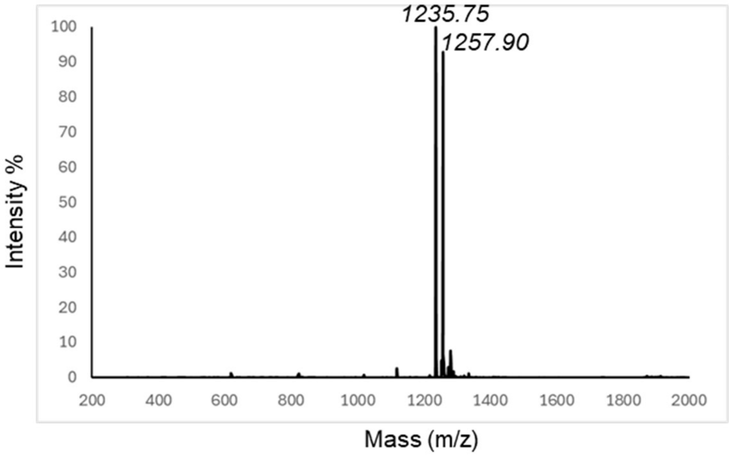

Calculated for $C_{69}H_{90}N_{10}O_{11}$	
$[M+H]^+$	1235.69
$[M+Na]^+$	1257.67

Figure S1. (Continued)

Hum W amide (**15**)

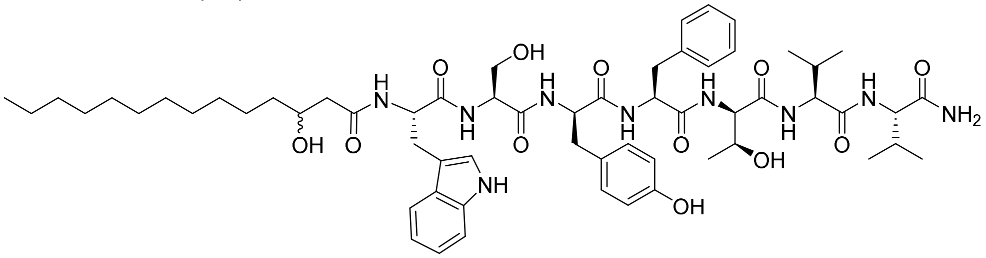

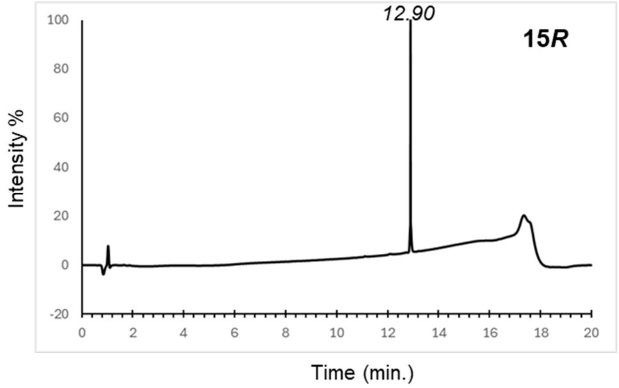

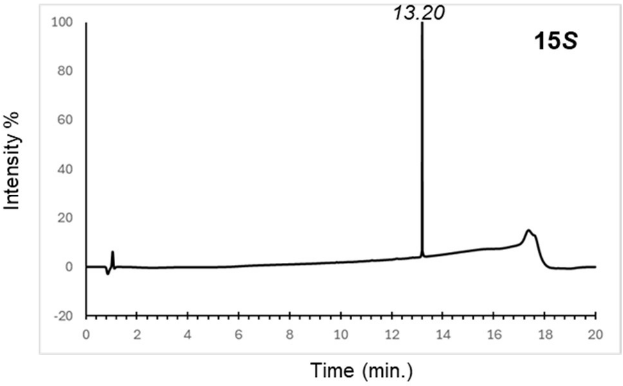

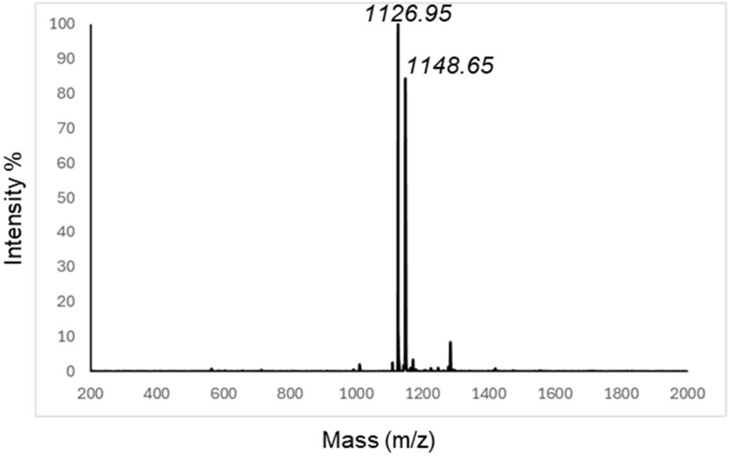

Calculated for $C_{60}H_{87}N_9O_{12}$	
$[M+H]^+$	1126.66
$[M+Na]^+$	1148.64

Figure S1. (Continued)

Hum W1,3 amide (**16**)

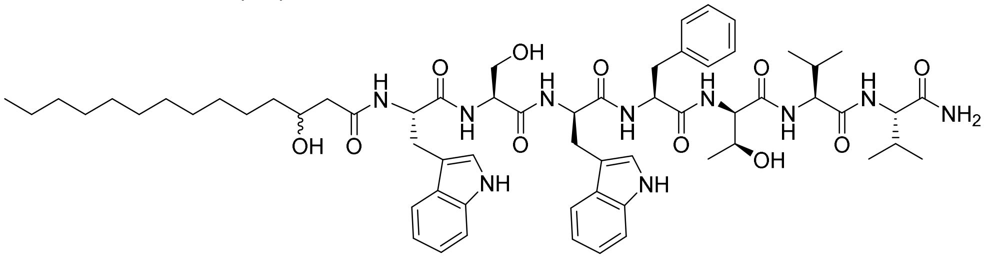

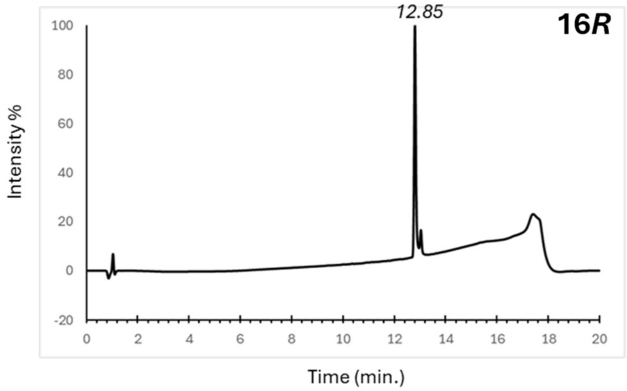

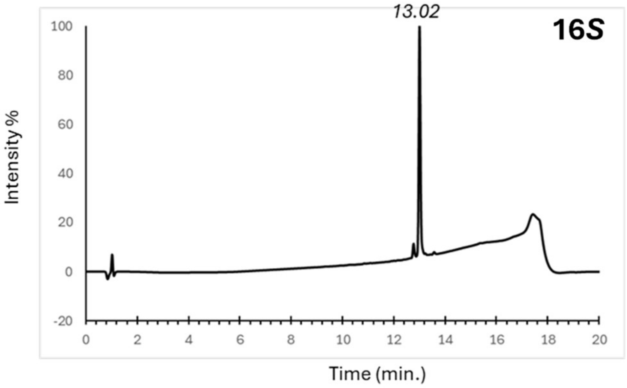

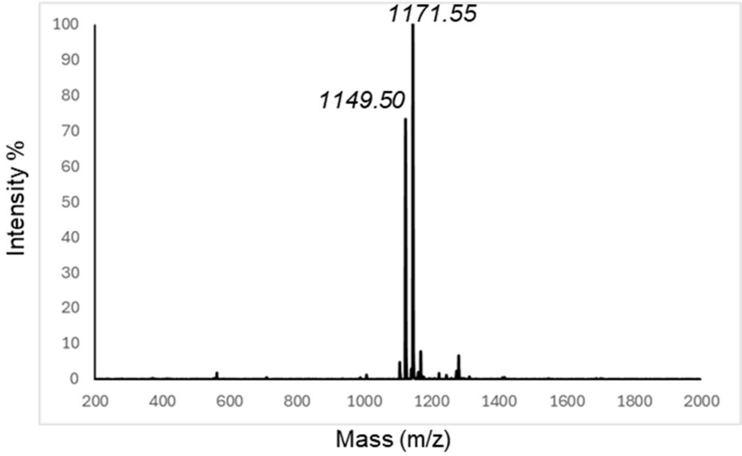

Calculated for $C_{62}H_{88}N_{10}O_{11}$	
$[M+H]^+$	1149.67
$[M+Na]^+$	1171.65

Figure S1. (Continued)

Hum W1,3-A6 amide (**17**)

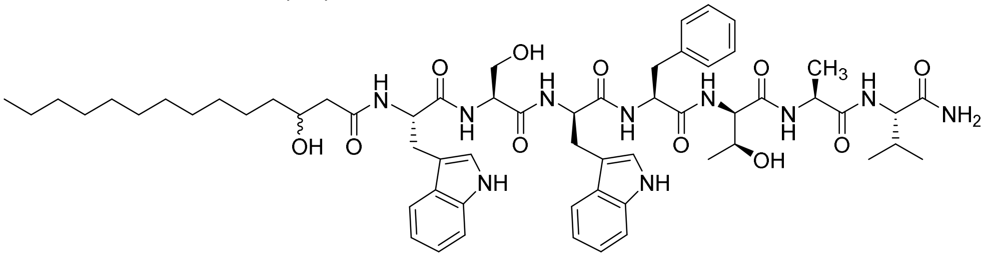

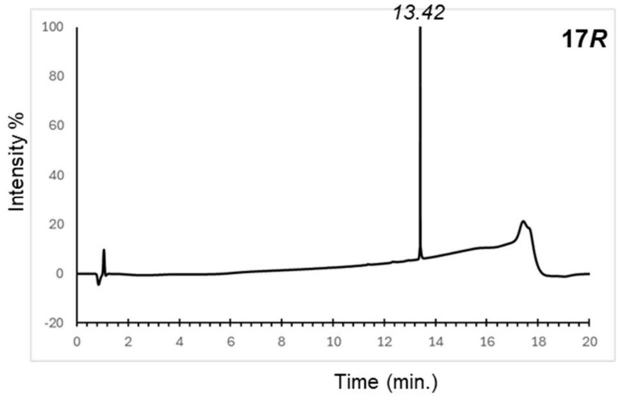

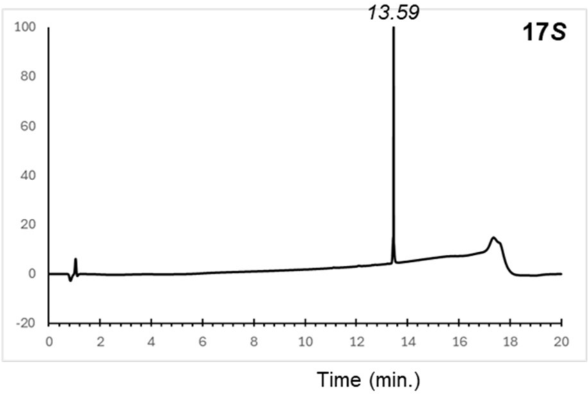

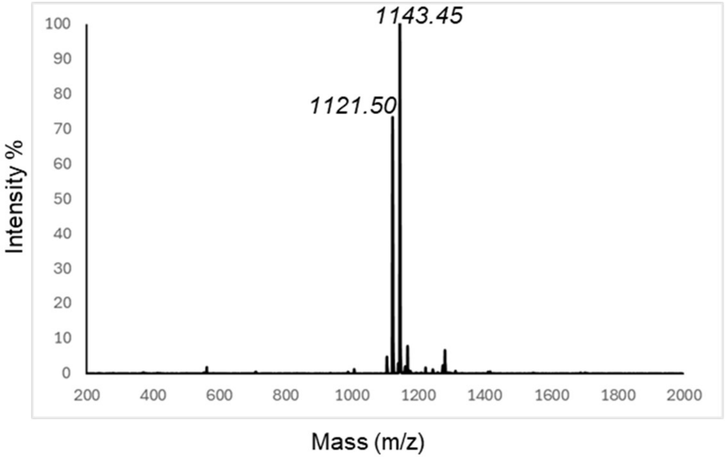

Calculated for $C_{60}H_{84}N_{10}O_{11}$	
$[M+H]^+$	1121.64
$[M+Na]^+$	1143.62

Figure S2. Agar diffusion assay of synthesized peptides **5-11** and **14-17** against *S. aureus* ATCC 29213

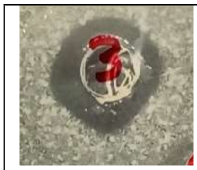	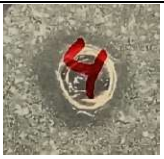	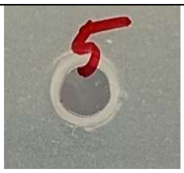	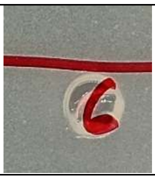	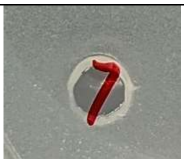
**1*R***	**1*S***	**5**	**6**	**7**
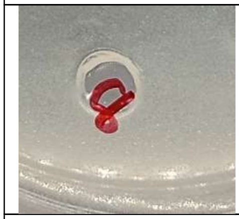	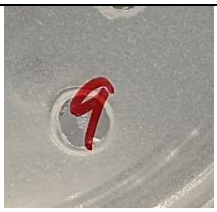	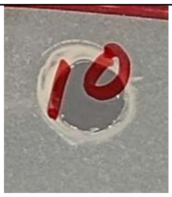	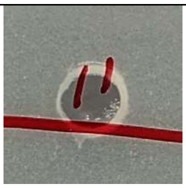	
**8**	**9**	**10**	**11**	
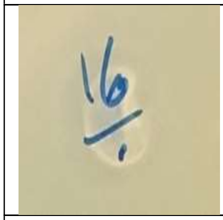	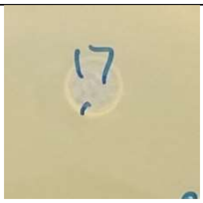	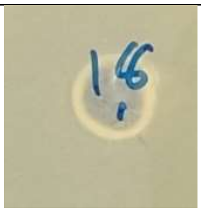	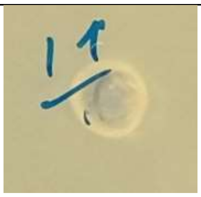	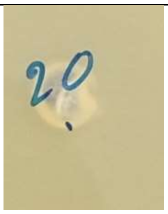
**14*R***	**14*S***	**15*R***	**15*S***	**16*R***
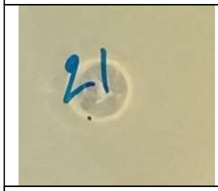	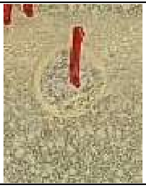	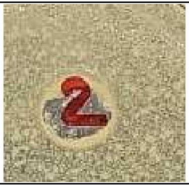		
**16*S***	**17*R***	**17S**		

Figure S3. Reference control: Tween-20. The CMC is the concentration at the intersection of the linear fits to the fluorescence intensity against concentration.

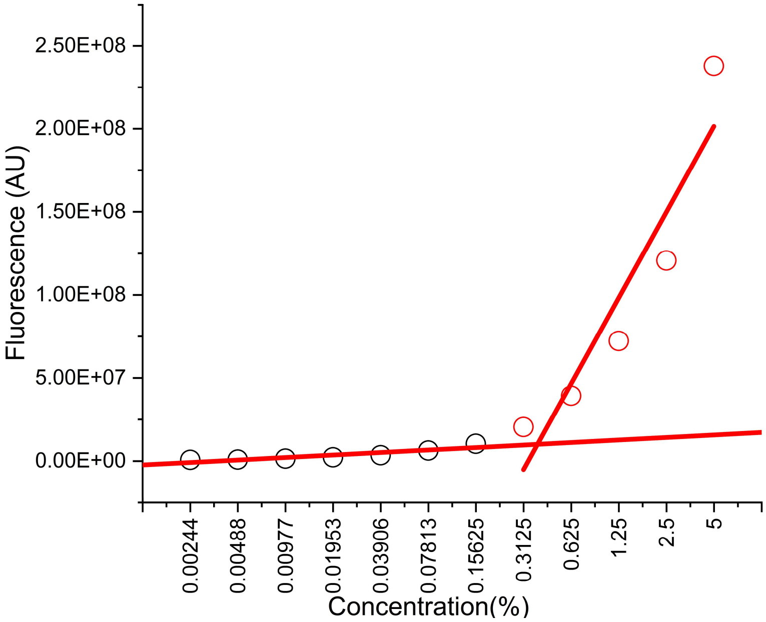

Figure S4. Molecular dynamics (MD) simulation of the Apo-MurJ (PDB ID: 5t77) protein over a 100 ns trajectory. Apo-Protein RMSD (A), Apo-Protein RMSF (B).

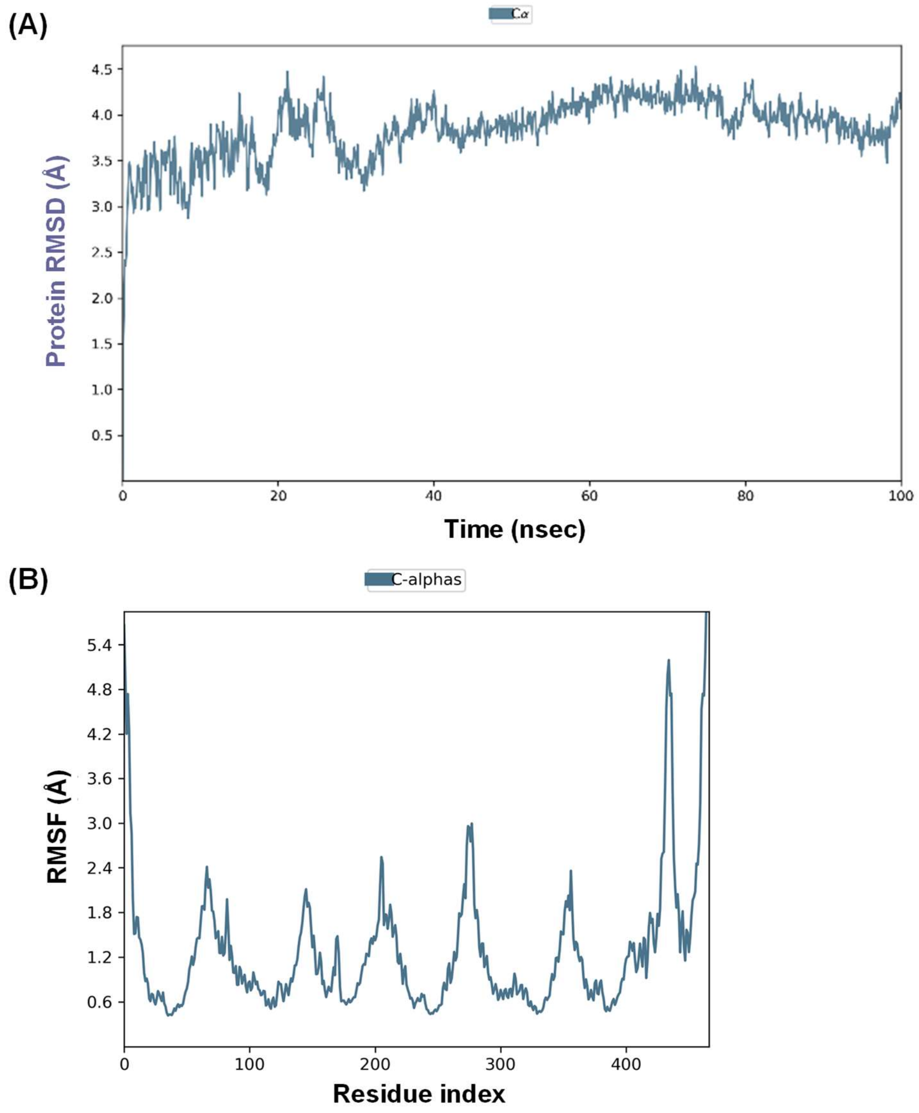

CASTp Analysis of MurJ (PDB ID: 5t77)
We utilized the CASTp 3.0 server to identify the potential binding pockets of MurJ (PDB ID: 5t77). This analysis was performed using a probe radius of 1.4 Å, which corresponds to the size of a dummy water molecule to accurately map the accessible surface pockets.

The results of the CASTp analysis, including pocket ID, surface area (SA), and volume (SA), are summarized in the table below:

Table S2. CASTp analysis, including pocket ID, surface area (SA), and volume (SA)*

Pocket ID	Area (SA) (Å^2)	Volume (SA) (Å3)
1	3030.301	5198.686
2	326.552	560.92
3	194.759	99.503
4	84.512	39.141
5	31.731	25.536
6	47.486	20.434
7	29.787	15.29
8	30.291	11.089
9	18.983	7.617
10	30.207	6.699
11	15.692	5.682

*Pocket ID 12 to 41 gave volumes < 2 Å^3

Key Findings from CASTp Analysis

- Pocket 1 is the largest, with an area of 3030.301 Å^2 and a volume of 5198.686 Å^3. This pocket is likely to be the primary binding site for potential ligands.
- Pocket 2 is significantly smaller, with a volume of 560.92 Å^3, and could represent a secondary interaction site.
- Pockets 3 to 10 exhibit progressively smaller sizes and may represent additional potential ligand interaction sites or solvent-accessible cavities.

Table S3. Key residues associated with each binding pocket of MurJ (PDB ID: 5t77)

Pocket ID	Annotation	Chain	Residue Positions	Description	Sequence	Binding Sites
1	Topological domain	A	24-35	Periplasmic	RDVLFAKYFGVS	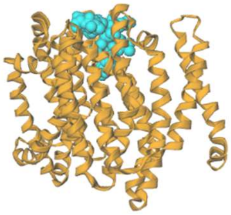
2	Transmembrane region	A	36-56	Helical	YELDAYFIAIMFPFFLRKVFG	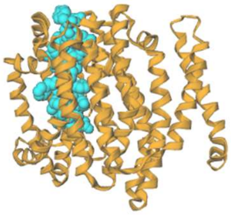
3	Topological domain	A	57-78	Cytoplasmic	EGAMSSAFVPLYSEKSGEEKDK	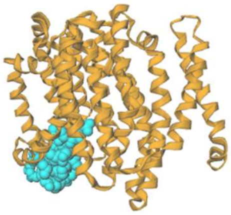
4	Transmembrane region	A	79-99	Helical	FLSSVINGFSLIILALVILSY	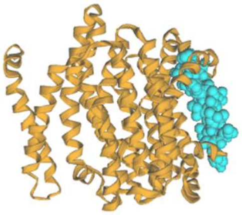
5	Topological domain	A	100-123	Periplasmic	FFPELIINLFGAGSSHETKILAKK	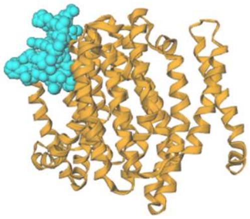
6	Transmembrane region	A	124-144	Helical	LLLITSPSIYFIFLWAISYSI	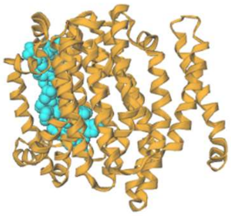

7	Topological domain	A	145-150	Cytoplasmic	LNTNNK	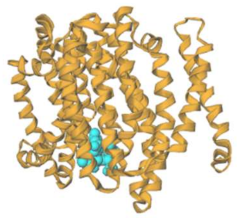
8	Transmembrane region	A	151-171	Helical	FFWPALTPSISNITIIIGTFL	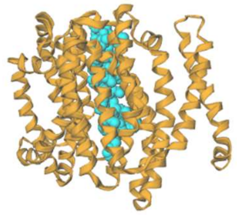
9	Topological domain	A	172-175	Periplasmic	STKY	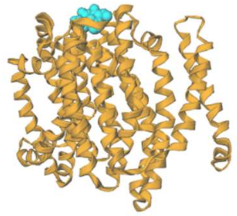
10	Transmembrane region	A	176-196	Helical	GIISPTIGFLIGSILMFFSII	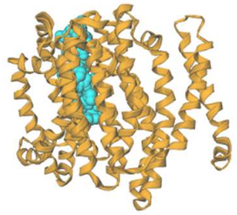

These results provide insights into the structural features of MurJ, highlighting potential binding sites that may be important for ligand interactions.

Correlation of CASTp and Docking Results

The docking analysis of ligands revealed significant interactions with key residues identified in the CASTp analysis. The **13*S*** ligand, which exhibited the highest binding affinity (-13.0585 kcal/mol), interacted with critical residues such as Arg18, Gly262, and Tyr261, which were also identified as major contributors in the CASTp pocket analysis.

The strong correlation between CASTp-predicted pocket residues and docking interactions validates the computational approach and suggests that these ligands could be promising candidates for targeting MOP flippase MurJ. The docking results provide further insight into the binding preferences of the ligands, supporting their potential role in inhibiting the function of MurJ by interacting with critical active site residues.

Table S4. Binding energy values of the five best binding poses predicted by Autodock Vina.

Ligands	12R	12S	13R	13S
Binding Energy (Kcal/mol)	-12.8998	-11.8257	-12.5367	-13.0585
	-12.056	-11.787	-11.9046	-12.645
	-11.9555	-11.6929	-11.3153	-12.6226
	-11.8006	-11.5555	-11.2711	-12.2223
	-11.5145	-11.1119	-10.8034	-11.231
